# Enhanced Near‐Infrared Photocatalytic Eradication of MRSA Biofilms and Osseointegration Using Oxide Perovskite‐Based P–N Heterojunction

**DOI:** 10.1002/advs.202002211

**Published:** 2021-06-19

**Authors:** Congyang Mao, Weidong Zhu, Yiming Xiang, Yizhou Zhu, Jie Shen, Xiangmei Liu, Shuilin Wu, Kenneth M. C. Cheung, Kelvin Wai Kwok Yeung

**Affiliations:** ^1^ Department of Orthopaedics and Traumatology Li Ka Shing Faculty of Medicine The University of Hong Kong Pokfulam Hong Kong 999077 China; ^2^ Biomedical Materials Engineering Research Center Collaborative Innovation Center for Advanced Organic Chemical Materials Co‐constructed by the Province and Ministry Hubei Key Laboratory of Polymer Materials Ministry‐of‐Education Key Laboratory for the Green Preparation and Application of Functional Materials School of Materials Science and Engineering Hubei University Wuhan 430062 China; ^3^ School of Materials Science and Engineering the Key Laboratory of Advanced Ceramics and Machining Technology by the Ministry of Education of China Tianjin University Tianjin 300072 China; ^4^ Shenzhen Key Laboratory for Innovative Technology in Orthopaedic Trauma Department of Orthopaedics and Traumatology The University of Hong Kong‐Shenzhen Hospital Shenzhen 518053 China

**Keywords:** charge transfer, MRSA biofilm, osseointegration, photocatalytic, P–N heterojunction

## Abstract

Methicillin‐resistant *Staphylococcus aureus* (MRSA) biofilm infections after orthopedic implant increase the risk of failure and potentially cause amputation of limbs or life‐threatening sepsis in severe cases. Additionally, satisfactory bone‐implant integration is another important indicator of an ideal implant. Here, an antibiotic‐free antibacterial nanofilm based on oxide perovskite‐type calcium titanate (CTO)/fibrous red phosphorus (RP) on titanium implant surface (Ti‐CTO/RP) in which the P–N heterojunction and internal electric field are established at the heterointerface, is designed. Near‐infrared light‐excited electron–hole pairs are effectively separated and transferred through the synergism of the internal electric field and band offset, which strongly boosts the photocatalytic eradication of MRSA biofilms by reactive oxygen species with an efficacy of 99.42% ± 0.22% in vivo. Additionally, the charge transfer endows the heterostructure with hyperthermia to assist biofilm eradication. Furthermore, CTO/RP nanofilm provides a superior biocompatible and osteoconductive platform that enables the proliferation and osteogenic differentiation of mesenchymal stem cells, thus contributing to the subsequent implant‐to‐bone osseointegration after eradicating MRSA biofilms.

## Graphical abstract

Driven by the synergism of internal electric field and band offset after alignment of the Fermi energy at the heterointerface, near‐infrared light excited electron‐hole pairs are effectively separated and transferred, which facilitates the generation of reactive oxygen species and heat to synergistically eradicate methicillin‐resistant *Staphylococcus aureus* biofilm. Additionally, upregulated osteogenic related genes promote the osseointegration.

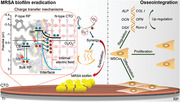

## 1 Introduction

Titanium (Ti)‐based alloys have been extensively used in orthopedic implants since the early 1970s because of their low density and elasticity modulus, excellent biocompatibility, and benign mechanical properties.^[^ ^1^ ^]^ However, *Staphylococcus aureus* (*S. aureus*), which is one of commonly seen pathogens, always induces implants‐associated infections that results in delayed postoperative bone tissue repair process, implant removal due to failure of surgery, and even amputation or life‐threatening sepsis in severe cases.^[^ ^2^ ^]^ Once the infection happens, it usually motivates a conventional long‐term antibiotic treatment in order to combat infections.^[^ ^3^ ^]^ Repeated antibiotic therapy is extremely easy to induce drug‐resistant bacteria,^[^ ^4^ ^]^ and more than 40% of drug‐resistant *S. aureus* bacteria are methicillin‐resistant *S. aureus* (MRSA).^[^ ^5^ ^]^ It has clearly become a global healthcare challenge associated with orthopedic implant infections.^[^ ^6^ ^]^ More seriously, MRSA biofilms develop preferentially on the surface of implants once the timely and effective treatment is not available, which further increases the difficulty of treatment due to strong abilities of MRSA biofilms to withstand host immune responses and inhibit the penetration of bactericide, especially for antibiotics.^[^ ^2^, ^7^ ^]^ Therefore, the exploration of antibiotic‐free therapeutic platform to combat MRSA biofilms‐induced orthopedic implant infections rapidly and efficiently has become imperative. Currently, most of implants optimizations mainly focus on the promotion of biocompatibility and bone regeneration by surface modification with biocompatible coatings or nanoscale disordered structures that favor osteoblasts proliferation and differentiation.^[^ ^8^ ^]^ However, the antimicrobial properties of implants are commonly neglected because biocompatibility and self‐antimicrobial properties are antithetic. Therefore, exploring an advanced bifunctional system to simultaneously eliminate the established MRSA biofilms and promote osseointegration resulting in bone healing is still challenging.

Calcium titanate (CTO), an important bioactive oxide perovskite ceramic, can provide a calcium source for bone regeneration after coating on the surface of implant. And CTO exhibits better osteogenic potential as compared with other oxide perovskites, including strontium titanate and barium titanate.^[^ ^9^ ^]^ Importantly, pure CTO is not normally ferroelectric at any temperature due to its highly symmetric orthorhombic crystal structure, which makes it more suitable for bone implant material.^[^ ^10^ ^]^ In addition, CTO as a photocatalytic candidate not only showed better stability and biocompatibility compared with other halide perovskites, but also can achieve photocatalysis that assists the release of reactive oxygen species (ROS) to kill bacteria under the irradiation of ultraviolet (UV).^[^ ^9^, ^11^ ^]^ In a typical photocatalytic process, electrons and holes induced by electron transition and charge transfer participate in redox reactions and produce target products, including ROS,^[^ ^12^ ^]^ while inefficient separation and transfer of electron–hole pairs is detrimental to the photocatalytic process of CTO related to its practical antibacterial effects.^[^ ^13^ ^]^ More seriously, UV well‐known carcinogenicity limits its application in medicine.^[^ ^14^ ^]^ Recently, researchers have modified CTO with platinum^[^ ^15^ ^]^ and silver^[^ ^16^ ^]^ to enhance the photocatalytic performance of CTO under UV and even visible light irradiation. However, both UV and visible light cannot effectively penetrate tissue for bone implant therapy.^[^ ^17^ ^]^ By contrast, near‐infrared light (NIRL) at wavelengths of 808 nm is often used in biomedicine due to its powerful penetration ability (several centimeters) into biological tissue with low absorption and scattering coefficients among all light sources.^[^ ^18^ ^]^ However, it is impossible for CTO to perform the photocatalytic antibacterial behavior under 808 nm NIRL irradiation because the energy gap (more than 3.0 eV) of CTO is much larger than the photonic energy (1.53 eV) of 808 nm NIRL resulting in the failure of electron transition.^[^ ^19^ ^]^ Therefore, achieving photocatalytic antibacterial effects of CTO under 808 nm NIRL by enhancing charge separation and transfer would bring a revolutionary breakthrough for the development of orthopedic implants.

**Scheme 1:**
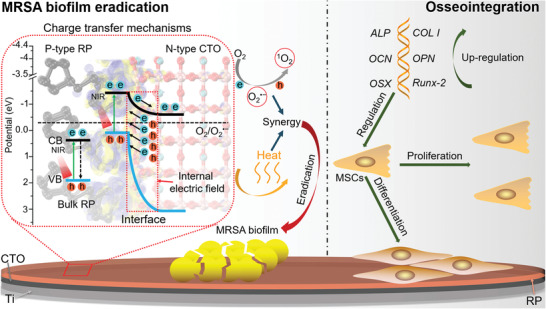
Schematic diagram of enhanced NIRL photocatalytic eradication of MRSA biofilms and osseointegration of CTO/RP P–N heterojunction. Driven by the synergism of internal electric field and band offset after alignment of the Fermi energy at the heterointerface, NIRL excited electron–hole pairs are effectively separated and transferred, which facilitate the generation of ROS and heat to synergistically eradicate MRSA biofilm on the surface of Ti orthopedic implant. On the other hand, upregulated osteogenic related genes promoted the osteogenic differentiation ability of mesenchymal stem cells (MSCs).

The P–N heterojunction has been widely considered in the fields of electronics and optoelectronics in order to improve the efficiency of charge separation and transfer.^[^ ^20^ ^]^ It is feasible to introduce a P‐type semiconductor to be combined with N‐type CTO to accelerate the separation and transfer of photogenerated electron–hole pairs. In this study, we designed a P–N heterojunction nanofilm by introducing fibrous red phosphorus (RP) onto the surface of oxide perovskite‐type CTO. Owing to its lower bandgap (1.50 eV) than the energy provided by 808 nm NIRL photons (1.53 eV),^[^ ^19b^ ^]^ the single fibrous RP film can be inspired to generate electron–hole pairs after electron transition under 808 nm NIRL irradiation. However, no ROS are detected after irradiation under 808 nm NIRL. As shown in **Scheme** **1**, the band alignment from hybrid density functional theory (DFT) calculations shows that the potential of the conduction band (CB) edge position (0.37 eV) of pure RP is more positive than that of O_2_/O_2_^•−^ (−0.33 eV), versus the normal hydrogen electrode (NHE), thereby failing to reduce oxygen at the electron side.^[^ ^21^ ^]^ Therefore, charges cannot be efficiently transferred to participate in the reaction of ROS generation.

However, the construction of the P–N heterojunction facilitates the establishment of internal electric field and band offset after the valence/conduction band edge positions of the RP and CTO are shifted (Scheme 1), contributing to the separation and transfer of photogenerated electron–hole pairs. Additionally, band offset induces electron transfer from the CB of the RP to the CB of the CTO. Thus, oxygen reduction and ROS generation are observed because the potential of the CB edge position (−0.54 eV) of the CTO is more negative than that of O_2_/O_2_^•−^ (−0.33 eV). Furthermore, the charge transfer also induces deep‐level defects (DLDs), hot carrier relaxation and electron loops, which result in an excellent photothermal property. Therefore, this novel P–N type CTO/RP heterojunction can be endowed with enhanced photocatalysis and hyperthermia under 808 nm NIRL irradiation, thus rendering the heterojunction powerful for the eradication of mature MRSA biofilms under NIRL irradiation in rats’ MRSA biofilm infected orthopedic implant model. In vivo results further indicate that the superiorly biocompatible and osteo‐inductive platform induced by calcium and phosphorus sources enables the subsequent bone regeneration and osseointegration after eradicating MRSA biofilms. Collectively, a novel and bifunctional therapeutic platform is designed by constructing CTO/RP heterojunction to drive charge transfer‐guided efficient eradication of established MRSA biofilms and simultaneously induce osseointegration (Scheme 1), which can be an efficient modification strategy for ideal orthopedic implants.

## 2 Results and Discussion

**Figure 1:**
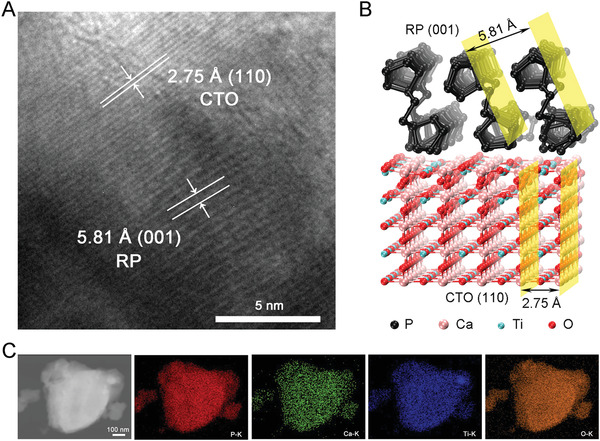
Morphology of CTO/RP P–N heterojunction. A) High‐resolution transmission electron microscopy (HRTEM) image of P–N heterointerface of CTO/RP and corresponding lattice spacing calibration of CTO and RP. Scale bar: 5 nm. B) Optimized geometric structures of CTO/RP P–N heterojunction, where black, pink, pale green, and red spheres represent P, Ca, Ti, and O atoms, respectively. The yellow plane refers to the corresponding lattice facets. C) Energy dispersive X‐ray spectroscopy (EDS) mapping of CTO/RP P–N heterojunction. (red: P; green: Ca; blue: Ti; orange: O)

Mechanically polished pure Ti plate was selected as the substrate with clear scratches (Figure S1, Supporting Information), and the nanofilm of CTO was first grown on the surface of Ti (Figure S2A, Supporting Information), which was termed Ti‐CTO. The high‐resolution transmission electron microscopy images of the nanofilm of the CTO showed clear (110) facets with parallel lattice fringes and a d‐spacing of 2.75 Å (Figure S2B,C, Supporting Information), which were consistent with typical perovskite‐type CTO (cubic phase).^[^ ^22^ ^]^ Additionally, atomic structures showed that CTO displayed the space group *Pm3m* [221] with a cubic perovskite structure (Figure S2D, Supporting Information). Subsequently, an RP nanofilm with a uniform distribution of particles was deposited on the Ti and Ti‐CTO substrates by chemical vapor deposition to obtain Ti‐RP (Figure S3A, Supporting Information) and Ti‐CTO/RP, respectively. The (001) facets with the parallel lattice fringe and a *d*‐spacing of 5.81 Å (Figure S3B,C, Supporting Information) was were observed, which was identified as fibrous phosphorus.^[^ ^23^ ^]^ The atomic structure of fibrous RP was the space group [*P_21_*] with a triclinic structure (Figure S3D, Supporting Information). The CTO/RP nanofilm exhibited a similar morphology to the CTO and RP nanofilms (Figure S4A,B, Supporting Information) and the corresponding lattice fringes and *d*‐spacing were observed (**Figure** **1A**). The (110) and (001) facets corresponded to the CTO and RP, respectively. The heteroepitaxial reaction between the CTO (110) and RP (001), as shown in Figure 1B, will be discussed below. The elemental mapping images (Figure 1C) of the heterostructures nanofilm also showed the uniform distribution of Ti, O, Ca, and P, further indicating the uniform distribution of the CTO and RP. The adhesion strength of CTO/RP coating as indicated by critical load in the scratch test was about 2.5 mN (Figure S5, Supporting Information), suggesting that the CTO/RP coating is not easily detached from the Ti surface as compared with others.^[^ ^24^ ^]^

**Figure 2:**
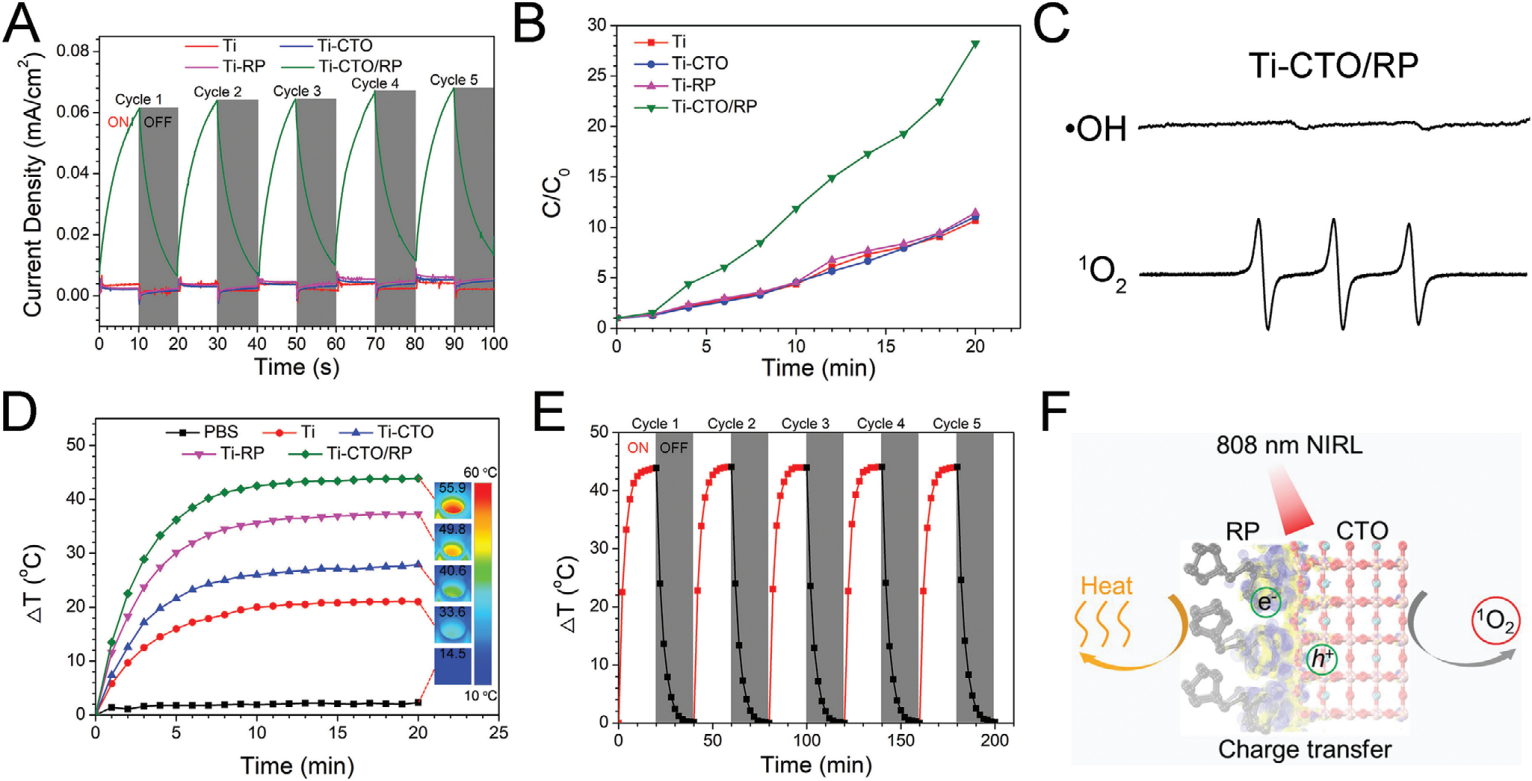
Photocatalytic performance and energy conversion under the NIRL irradiation. A) Transient photocurrent response plots of CTO/RP P–N heterojunction compared with those of Ti, CTO, and RP by electrochemical workstation through adopting 5 cycles of 808 nm NIRL on/off. B) The ROS generation versus time of CTO/RP P–N heterojunction compared with that of Ti, CTO, and RP shows the enhanced photocatalysis by CTO/RP heterojunction. C) The ESR spectra of •OH and three lines with the intensities of 1:1:1 (^1^O_2_) trapped by DMPO (0.1 m) and TEMP (50 × 10^−3^ m), respectively, after irradiated CTO/RP heterojunction with spin traps under NIRL for 20 min. D) Heating curves versus time comparison of CTO/RP and the other samples with NIRL irradiation show the enhanced photothermal ability of CTO/RP. And the inserted thermal images show the detected temperature of corresponding samples after NIRL irradiation for 20 min. E) Photothermal stability of CTO/RP by adopting 5 cycles of NIRL on/off. F) Schematic diagram of heat and ^1^O_2_ release under 808 nm NIRL irradiation.

Improving the separation and transfer of electron–hole pairs facilitates the participation of charges in the reaction of ROS generation,^[^ ^25^ ^]^ and the photocurrent is induced by charge transfer after electron–hole separation.^[^ ^26^ ^]^ Therefore, the photocurrent densities of Ti, CTO, RP, and CTO/RP were examined by adopting five cycles of 808 nm NIRL on/off by electrochemical workstation. As shown in **Figure** **2A**, after the samples were irradiated by 808 nm NIRL, the Ti‐CTO/RP generated obvious photocurrent density while the other groups generated almost no photocurrent, indicating that the enhancement of electron–hole separation and efficient charge transfer were successfully established. And electrochemical impedance spectroscopy showed that Ti‐CTO/RP had a decreased interface resistance (Figure S6, Supporting Information), indicating that charge transfer barrier of Ti‐CTO/RP was declined, which is beneficial to the transfer of photocarrier. Additionally, Ti‐CTO/RP exhibited a decreased photoluminescence intensity (Figure S7, Supporting Information), confirming the longer lifetime of photocarrier and effective separation of electron–hole pairs. Next, 2′,7′‐dichlorodihydrofluorescein diacetate (DCFH‐DA) was used to evaluate the ROS generation. After the DA molecule from DCFH‐DA was hydrolyzed by sodium hydroxide according to the manufacturer's instructions, the DCFH could be oxidized by ROS or oxygen to form the DCF. The detection of DCF could directly reflect the generation of ROS. The concentration of DCF in the Ti‐CTO/RP group was ≈28‐fold greater than the initial concentration after 808 nm NIRL irradiation for 20 min (Figure 2B), demonstrating the generation of ROS. Other groups exhibited almost the same increase (≈10‐fold) of DCF, which can be attributed to the fact that the oxygen in the solution also oxidized the DCFH to generate DCF. An electron spin resonance (ESR) spectrometer was used to identify the species of ROS. The detection of hydroxyl radicals (•OH) was ESR‐silent, whereas the spectrum showed the three lines (1:1:1) characteristic of the typical generation of singlet oxygen (^1^O_2_) (Figure 2C), indicating that the generation of ^1^O_2_ from the Ti‐CTO/RP was found after irradiation by NIRL. Additionally, Ti‐CTO/RP showed powerful NIRL photodegradation of 1,3‐diphenylisobenzofuran, which further reveals the generation of ⁠^1^O⁠_2_ by Ti‐CTO/RP under the irradiated of NIRL (Figure S8, Supporting Information).

A UV–vis–NIR absorbance spectrophotometer (300–1200 nm) was used to investigate the light absorption abilities of different samples at a wavelength of 808 nm, since the photothermal property of material is related to its light absorption ability. Higher absorbance indicated superior photothermal conversion.^[^ ^27^ ^]^ The Ti‐CTO/RP exhibited an obviously enhanced absorbance at a wavelength of 808 nm (Figure S9, Supporting Information). Thus, the photothermal tests on the Ti, CTO, RP, and CTO/RP were performed in 200 µL of phosphate buffer saline (PBS, pH = 7.4) solution under 808 nm NIRL for 20 min. The sample with PBS solution only served as the control. A thermal imager was applied to collect the thermal images and record the temperature. The red color in the thermal images represents a higher temperature, whereas the blue color indicates a lower temperature (Figure 2D; Figure S10, Supporting Information). Both the thermal images and temperature curves indicated that the Ti‐CTO/RP group showed excellent photothermal performance. The temperature in the group increased by 43.9 °C after 10 min of 808 nm NIRL irradiation and then stabilized, which was consistent with the UV–vis–NIR absorbance results. By contrast, the temperature of the PBS solution (control) remained unchanged during irradiation. After stimulation for 20 min, the suspension temperature of the Ti, CTO, RP, and CTO/RP were 33.6, 40.6, 49.8, and 55.9 °C, respectively. The temperature in the Ti‐CTO/RP group was much higher than in the other groups even though the irradiation time was the same. Moreover, the efficiency of suspension temperature variations above the Ti‐CTO/RP were recorded under 808 nm NIRL irradiation for 20 min, followed by turning off the laser and naturally cooling for another 20 min for a total of five cycles of laser on/off. The same phenomenon was observed during the five cycles of laser on/off as shown in Figure 2E, demonstrating the excellent photothermal stability. Collectively, CTO/RP showed enhanced release of heat and ^1^O_2_ by charge transfer under 808 nm NIRL irradiation (Figure 2F), which will be discussed below.

The hybrid DFT calculations were applied to understand the mechanisms of enhanced NIRL absorption and the photogenerated electrons transfer of the CTO/RP heterojunction. As shown in Figure 1A, the CTO (110) lattice facets were heteroepitaxially stacked on the RP (001) lattice facets; thus, the CTO (110), RP (001), and CTO (110)/RP (001) heterojunctions were chosen and constructed in DFT. The interface formation energy of the entire model CTO/RP interfaces was calculated as −78.40 eV. The negative value further identified the stable interfaces between the CTO (110) and RP (001).^[^ ^21c^ ^]^

**Figure 3:**
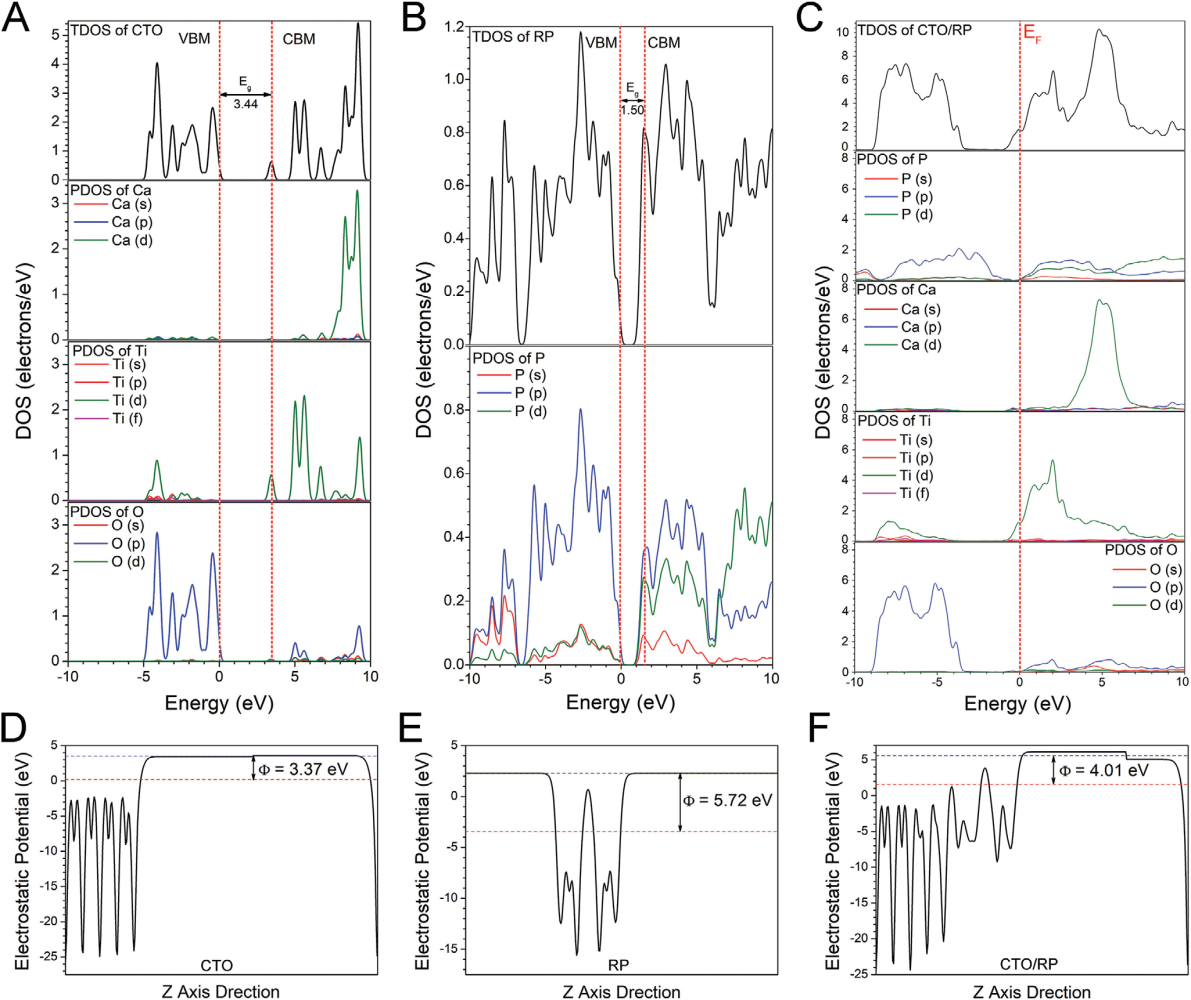
Band structures and work function of CTO, RP, and CTO/RP P–N heterojunction. A,B) Calculated TDOS and PDOS of (A) CTO and (B) RP, respectively. The VBM is selected as the Fermi energy level and set as 0 for reference. The energy gap between the VBM and CBM is identified as bandgap. And the calculated bandgap of CTO and RP is 3.44 and 1.50 eV, respectively. C) Calculated TDOS and PDOS of CTO/RP P–N heterojunction. The Fermi energy level is indicated by red vertical dashed line. D–F) Calculated electrostatic potentials of (D) CTO, (E) RP, and (F) CTO/RP P–N heterojunction, respectively. The red horizontal dashed line denotes the Fermi energy level, whereas the blue horizontal dashed line indicates the vacuum energy level. The electrostatic potential gap between the Fermi energy level and the vacuum energy level is identified as work function. And the calculated work function of CTO, RP, and CTO/RP P–N is 3.37, 5.72, and 4.01 eV, respectively.

First, the total density of state (TDOS) and project density of state (PDOS) of the CTO (110), RP (001) and CTO (110)/RP (001) heterojunction were calculated using the Heyd–Scuseria–Ernzerhof functional. The valence band maximum (VBM) was set at 0 and selected as the Fermi energy level for reference. The gap between the conduction band minimum (CBM) and the VBM was identified as the bandgap. Thus, the calculated bandgaps of the CTO and RP were 3.44 and 1.50 eV, respectively (**Figure** **3A**,B), which agreed with the previous calculated values.^[^ ^28^ ^]^ Additionally, the VBM of the CTO was predominantly composed of O 2p orbital, whereas the CBM was dominated by Ti 3d orbital. The VBM and CBM of the RP were mainly occupied by P 3p orbital. Importantly, the TDOS of the CTO/RP indicated more hybrid orbitals at the Fermi energy level (Figure 3C), which can be attributed to the interfacial reaction between the CTO and RP. The details will be discussed in the charge density difference. As a critical parameter, the work function was commonly used for band alignment.^[^ ^29^ ^]^ The work functions of the CTO, RP and CTO/RP were 3.37, 5.72, and 4.01 eV, respectively (Figure 3D–F). The higher work function of the RP demonstrated that the photogenerated electrons flowed from the CTO to the RP until the Fermi energy levels of the CTO and RP were aligned, which was further demonstrated by the charge density difference.

**Figure 4:**
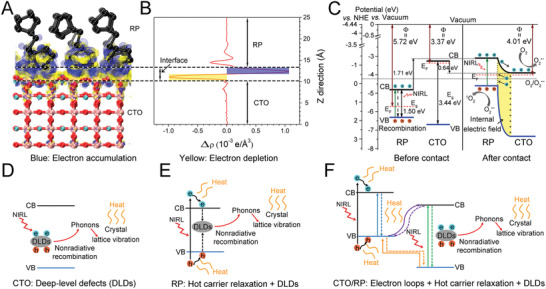
DFT calculation and enhanced photothermal mechanisms of CTO/RP P–N heterojunction. A) 3D charge density difference of CTO/RP P–N heterojunction, where black, pink, pale green, and red spheres represent P, Ca, Ti, and O atoms, respectively. B) Planar‐averaged electron density difference of CTO/RP P–N heterojunction quantifies about 1.0 × 10^−3^ electrons transferred at the interface. The blue and yellow areas represent electron accumulation and depletion, respectively. C) Diagram of band alignment of CTO/RP P–N heterojunction. Before contact, RP and CTO fail to show photocatalysis. Upon the NIRL (808 nm) irradiation, the electrons produced on the CB band edge of RP by the electron transition are unable to participate in the reduction of oxygen while CTO is unable to trigger electron transition. After contact, under the synergism of internal electric field and band offset, the enhancement of charge separation and electron transfer on the CB band edge of RP and CTO facilitates the charges to participate in photocatalysis. The yellow area indicates the internal electric field. D–F) Schematic illustration of photothermal mechanism (D) CTO, (E) RP, and (F) CTO/RP P–N heterojunction. Upon the NIRL (808 nm) irradiation, for CTO, some of the excited electrons and holes are captured by DLDs centers and then nonradiatively recombined to emit phonons, inducing intense crystal lattice vibration to release heat radiation; For RP, besides the DLDs, the electron–hole generation above the band edges and then relaxation to the band edges converts the extra energy into heat. For CTO/RP heterojunction, in addition to hot carrier relaxation and DLDs, the electron loops indicated by solid and dotted arrow pairs between CTO and RP convert the kinetic energy of electrons into heat.

As depicted in the 3D charge density difference (**Figure** **4A**), the blue and yellow regions represent the electron accumulation and depletion, respectively. The planar‐averaged electron density difference indicated that ≈1.0 × 10^−3^ electrons were transferred at the interface by calculus (Figure 4B). The negative and positive values at the interface indicated that the CTO side near the interface showed a depletion of electrons and carried positive charges, whereas the RP side near the interface exhibited an accumulation of electrons and carried negative charges. Thus, the charge redistribution at the interface revealed that the electrons flowed from the CTO part to the RP part, which was consistent with the results of work function. Importantly, the internal electric field was established at the interface by the accumulation of net charges on the CTO and RP sides, which was beneficial to the separation of electron–hole pairs.

The CB and valence band (VB) edge potentials were also essential for band alignment because the redox ability was determined by the positions of the CB and VB.^[^ ^21c^ ^]^ As shown in Figure 4C, based on the calculated bandgap, the CB and VB edge potentials of the RP were 0.37 and 1.87 eV versus the NHE, respectively, and the CB and VB edge potentials of the CTO were −1.18 and 2.26 eV, respectively. Before contact, the RP was a typical P‐type semiconductor when the Fermi energy level was close to the VB, while the CTO was an N‐type semiconductor with the Fermi energy level near the CB.^[^ ^30^ ^]^ It was noted that the narrow bandgap (1.50 eV) of the RP was lower than the energy of 808 nm NIRL photons (1.53 eV),^[^ ^19b^ ^]^ which endowed the RP with the abilities of electron transition and the subsequent generation of photoelectron–hole pairs under 808 nm NIRL irradiation. However, the RP failed to produce redox product (ROS) after irradiation under 808 nm NIRL (Figure 2B). As shown in Figure 4C, band alignment showed that the CB edge position of the RP was 0.37 eV (vs NHE), which is more positive than that of O_2_/O_2_^•−^ (−0.33 eV), thereby resulting in the failure of ROS generation through oxygen reduction.^[^ ^21^ ^]^ However, the RP and CTO shared an equalized Fermi energy level when the two semiconductors were in close contact, which significantly contributed to the shift in the relative positions of the VB and CB. Thus, the VB and CB edge potentials of the RP shifted upward by 1.71 eV, whereas the VB and CB edge potentials of the CTO shifted downward by 0.64 eV, as depicted in Figure 4C. Additionally, the position of the CB of the RP was higher than that of the CTO, which formed the band offset and contributed to the separation and transfer of photoinduced electrons from the CB of the RP to the CB of the CTO. The internal electric field (yellow region) also facilitated the migration of photoinduced electrons. Therefore, under the synergy of internal electric field and band offset, photogenerated electron–hole pairs were effectively separated and transferred, which was the typical character of a P–N heterojunction.^[^ ^31^ ^]^ Importantly, the CB position of the CTO changed to −0.54 eV, which was more negative than that of O_2_/O_2_^•−^ (−0.33 eV), thereby reducing oxygen to O_2_^•−^, as indicated in Equation (1).^[^ ^12a^ ^]^ Based on the charge conservation, the consumption of holes occurred through the reaction of ^1^O_2_ generation, as indicated in Equation (2).^[^ ^12a^ ^]^ Namely, the O_2_^•−^ was oxidized by the holes on the VB of the RP to generate ^1^O_2_, which was in good agreement with the results of the ROS identification (Figure 2C)

(1) $$O_{2}+e^{-}\to {O_{2}}^{•-}$$

(2) $${O_{2}}^{•-}+h^{+}\to^{1}O_{2}$$

The charge transfer theory was also used to understand the underlying mechanisms of enhanced photothermal performance after the formation of the P–N heterojunction. According to the Shockley–Read–Hall recombination theory, there are many DLDs, particularly in broad‐bandgap semiconductors with low carrier density.^[^ ^32^ ^]^ CTO is a typical broad‐bandgap semiconductor; thus, the DLDs in the CTO worked as the workstations for the nonradiative recombination of photoinduced electron–hole pairs to emit phonons (Figure 4D).^[^ ^32^ ^]^ Therefore, for the CTO, some of the excited electrons and holes were captured and nonradiatively recombined by DLDs to induce intense crystal lattice vibration by the emission of phonons and release heat radiation as a result of energy conservation. For the RP with a narrow bandgap, the energy of 808 nm NIRL photons was higher than the bandgap of RP, contributing to the formation of above‐bandgap electrons and holes, which then rapidly relaxed to the CB and VB edges, converting the irradiative energy into heat as a result of energy conservation (Figure 4E).^[^ ^33^ ^]^ Moreover, electron transfer was inhibited as indicated in band alignment (Figure 4C); thus, some of the excited electrons and holes were also captured by DLDs in the RP to generate heat. For the CTO/RP heterojunction, in addition to hot carrier relaxation and DLDs, there were many more electron loops (solid and dotted arrow pairs) between the CTO and RP for electron motion, which was favorable for enhancing the energy conversion based on the energy conservation (Figure 4F).^[^ ^34^ ^]^

To evidence the photocatalytic bacterial inactivation of CTO/RP coating, we built an in vitro antibacterial model under 808 nm NIRL irradiation. After treated the bacterial suspension with samples under 808 nm NIRL (0.5 W cm^−2^) or in the darkness for 20 min, bacterial suspension was then collected and spread on the Luria–Bertani (LB) agar plates to form the visible colony units (Figure S11, Supporting Information). The number of colony units was further calculated and the corresponding colony‐forming units (CFU) of bacterial suspension treated by different samples were obtained (Figure S12A,B, Supporting Information). For both *Escherichia coli* (*E. coli*, Gram‐negative, ATCC 25922) and MRSA (Gram‐positive, ATCC 43300), after in the darkness for 20 min, bacterial suspensions in all the groups kept almost the same concentration (above 10^7^ CFU mL^−1^), which was the initial concentration of the bacterial suspension, indicating no bactericidal effect of the component itself. However, the bacterial suspension in Ti‐CTO/RP decreased from 10^7^ to 10^5^ CFU mL^−1^ for both *E. coli* and MRSA after irradiated under 808 nm NIRL for 20 min, and the corresponding antibacterial ratios were calculated (Figure S13A,B, Supporting Information). Ti‐CTO/RP killed 99.61% ± 0.05% of *E. coli* and 99.78% ± 0.06% of MRSA after irradiated under 808 nm NIRL for 20 min. After five cycles of MRSA culture in high concentration were completed, the Ti‐CTO/RP was still able to kill more than 99% of MRSA (Figure S14, Supporting Information), indicating that the antibacterial potential of CTO/RP heterojunction was repeatable and reliable. The dominant mechanism of the enhanced bacterial killing abilities was due to the photocatalytic product (ROS) and the photothermal effect during 808 nm NIRL irradiating. The Ti‐CTO and Ti‐RP groups also killed small amounts of bacteria, which can be attributed to the moderate photothermal effect under 808 nm NIRL.

The morphologies and membrane integrities of bacteria after treated by different samples were further evaluated by a scanning electron microscope (SEM). *E. coli* showed the normal morphologies with smooth and typical rod shape while MRSA exhibited the normal morphologies with smooth and typical spherical shape after treated by all the samples in the darkness for 20 min (Figure S15, Supporting Information). By contrast, Ti‐CTO/RP caused serious damage to the bacteria after irradiation for 20 min as indicated by the red arrows. *E. coli* exhibited the distorted and wrinkled membranes while MRSA displayed the huge lesions and even be cracked after treated by Ti‐CTO/RP after irradiation under 808 nm NIRL, which was in good agreement with the photocatalytic bacterial inactivation results. The slight wrinkled membranes were also observed for both *E. coli* and MRSA in Ti‐CTO and Ti‐RP groups after irradiation due to the moderate photothermal effect. However, the *E. coli* and MRSA in pure Ti group kept the normal bacterial morphologies after irradiation by 808 nm NIRL. In addition, TEM was further applied to precisely examine the morphology of MRSA bacterial membrane. The MRSA bacteria on Ti group exhibited normal morphology and the membrane integrity maintained (Figure S16, Supporting Information). However, the bacterial membrane of MRSA bacteria on Ti‐CTO/RP group was seriously ruptured (red arrow) after irradiation.

To further reveal the in vitro MRSA biofilm eradication property of CTO/RP heterojunction, the MRSA suspension was cultured on the surface of Ti and Ti‐CTO/RP for 3d that allowed the formation of mature MRSA biofilm.^[^ ^35^ ^]^ After treated with 808 nm NIRL, the integrity of MRSA biofilm on Ti control sample remained unchanged in which the integrated cell membrane was found (Figure S17A, Supporting Information). By contrast, a high amount of ROS and hyperthermia condition created by CTO/RP coating on Ti surface ruptured the MRSA biofilm. Furthermore, with the results of live and dead staining on MRSA bacteria (Figure S17B, Supporting Information), green stain was observed on the Ti control, indicating that the MRSA biofilm was alive. However, the fluorescent image on the Ti‐CTO/RP group was completely red that represented the death of bacteria in MRSA biofilm.

**Figure 5:**
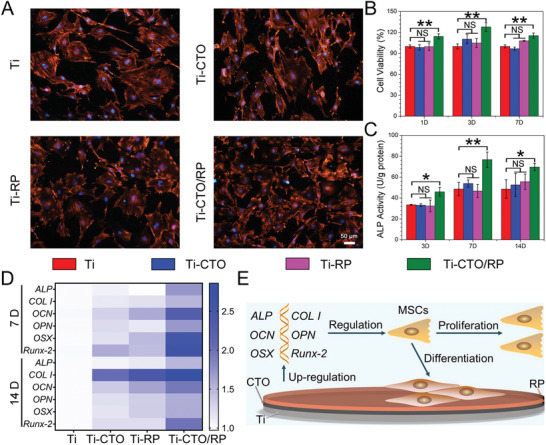
In vitro MSCs modulation. A) The fluorescence images of MSCs cultured on Ti, CTO, RP, and CTO/RP P–N heterojunction for 24 h after nucleus was stained with DAPI (blue) and F‐actin was stained with TRITC (bright orange). Scale bar: 50 µm. B) The viabilities of MSCs after cultured with Ti, CTO, RP, and CTO/RP P–N heterojunction for 1, 3, and 7 days. C) The ALP activities of MSCs after cultured with Ti, CTO, RP, and CTO/RP P–N heterojunction for 3, 7, and 14 days. D) The heat map reveals that the quantification of the bone related genes expression (*ALP*, *COL I*, *OCN*, *OPN*, *OSX*, and *Runx‐2*) in MSCs by qRT‐PCR using GAPDH as a reference gene after cultured with Ti, CTO, RP, and CTO/RP P–N heterojunction for 7 and 14 days. E) Schematic diagram of enhanced proliferation and differentiation of MSCs by upregulating bone related genes expression. Error bars indicate means ± standard deviations (*n* = 3): *p < 0.05, **p < 0.01, ***p < 0.001 (*t*‐test). NS: not significant (*P* > 0.05).

The in vitro cellular response of different samples was performed prior to in vivo animal experiments. The mesenchymal stem cells (MSCs) fluorescence images showed that the MSCs grew well on the surface of all samples (**Figure** **5A**), indicating the excellent biocompatibility of Ti, Ti‐CTO, Ti‐RP, and Ti‐CTO/RP. However, the more spindle‐like and elongated morphologies of MSCs were observed in Ti‐CTO/RP. The MSCs viabilities on the surface of Ti, Ti‐CTO, Ti‐RP, and Ti‐CTO/RP were further evaluated after culturing for 1, 3, and 7 days. As shown in Figure 5B, there were no significant differences in MSCs viabilities for Ti, Ti‐CTO, and Ti‐RP groups. However, Ti‐CTO/RP group showed a slight increase in MSCs viability, which was consistent with the results of cells fluorescence. The MSCs alkaline phosphatase (ALP) activities of different samples were then investigated after culturing for 3, 7, and 14 days. For a process that promoted osteogenic differentiation, ALP activity increased at earlier stage and then peaked at about 7 days, followed by the decline because ALP was a widely recognized biochemical marker of early osteogenic differentiation period. As shown in Figure 5C, there were no significant differences in ALP activities for Ti, Ti‐CTO, and Ti‐RP groups, and the ALP activities in these three groups basically remained unchanged as the time, indicating almost no osteogenic differentiation of MSCs during culturing. However, the ALP activities in Ti‐CTO/RP group were higher than that of in other groups after culturing for 3, 7, and 14 days. Moreover, ALP activities in Ti‐CTO/RP group first increased to peak at 7 days and then dropped after 14 days of incubation, demonstrating enhancement of osteogenic differentiation in Ti‐CTO/RP group. The dominant mechanism of the enhanced ALP activities was that the generated calcium phosphate (CAP) promoted osteogenic differentiation. And new CAP was produced by the combination of calcium ions (Ca^2+^) and phosphate anions (PO_4_^3−^) released from CTO and RP, respectively, as indicated in Equations (3) and (4)^[^ ^36^ ^]^

(3) $$P+O_{2}+H_{2}O\to H^{+}+{\mathrm{PO}}_{4}^{3-}$$

(4) $${\mathrm{PO}}_{4}^{3-}+Ca^{2+}+H_{2}O\to \mathrm{CAP}$$

To further understand the underlying mechanisms of promoted osteogenic differentiation, the expression of bone related genes in MSCs after culturing with Ti, Ti‐CTO, Ti‐RP, and Ti‐CTO/RP for 7 and 14 days were evaluated by quantitative real‐time polymerase chain reaction (qRT‐PCR). *ALP* was a commonly recognized biochemical marker in the early osteogenic differentiation period. As a novel zinc finger specific transcription factor, *Osterix* (*OSX*) was essential for cell proliferation and differentiation as well as bone generation. And *runt‐related transcription factor 2* (*Runx‐2*) was known as a crucial osteogenic transcription factor and was always served as the central control gene. In addition, *Runx‐2* was essential for the activation of downstream osteogenic genes, including *ALP*, *Collagen type I* (*COL I*), *Osteocalcin* (*OCN*), and *Osteopontin* (*OPN*), which were responsible for the formation of bone specific matrix proteins.^[^ ^37^ ^]^ In this study, Ti‐CTO/RP obviously upregulated the expression of *ALP*, *COL I*, *OCN*, *OPN*, *OSX*, and *Runx‐2* (Figure 5D; Figure S18, Supporting Information). Collectively, CTO/RP showed enhanced proliferation and differentiation of MSCs by upregulating bone related genes expression (Figure 5E), which further confirmed the potential of CTO/RP to promote osteogenesis.

**Figure 6:**
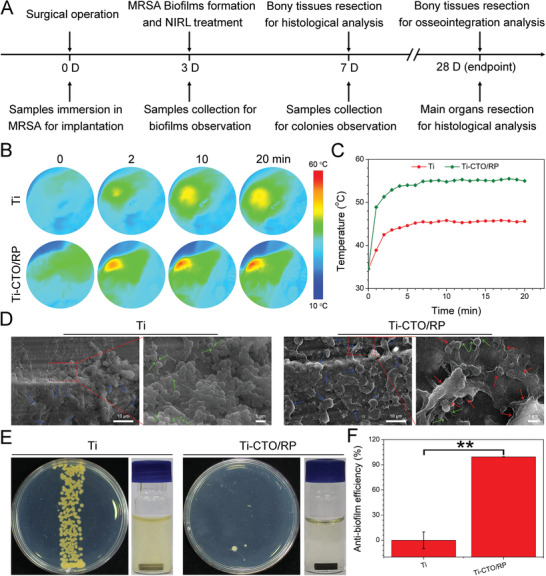
CTO/RP nanofilm for in vivo mature biofilm eradication. A) The treatment schedule for the rats’ MRSA biofilm infected orthopedic implant model. Samples were implanted into the intramedullary canal after immersed in MRSA for bacterial colony. After three days, MRSA biofilms were formed and treated by NIRL for SEM observation. After seven days, the therapeutic efficiency was determined by the observation of colony residue and histological analysis. At the endpoint of 28 days, the osteogenesis and bone‐implant osseointegration were determined by a µ‐CT system and biological safety was assessed by histological analysis. B) Thermal images at the implanted sites under NIRL (808 nm) irradiation for the eradication of established biofilms at three days postoperation. C) The corresponding heating curves versus time comparison of CTO/RP and Ti with NIRL irradiation shows the enhanced photothermal ability in vivo of CTO/RP. D) Representative SEM images of the MRSA biofilms on the surface of implants taken from rats at the end of treatments, where the blue arrows indicate the typical biofilms’ symbols of highly organized structures consisting of piles of colonies resembling mushrooms; the green arrows represent the bacterial exocrine polymers; while the red arrows indicate the severely deformed biofilms. Scale bar in low magnification images: 10 µm; Scale bar in high magnification images: 1 µm. E) The implants are taken from rats after the treatments for seven days; the formed MRSA biofilm colonies after the samples rolled several laps on the LB agar plates and then cultured at 37 °C for 48 h; and culture mediums after corresponding samples immersed in sterile LB broth at 37 °C for 24 h, where the turbid medium in Ti group evidences the live MRSA‐rich biofilms on the surface of sample while the transparent LB broth in CTO/RP group indicates the sterile implant surface. F) The corresponding anti‐biofilm efficiency of Ti and CTO/RP. Error bars indicate means ± standard deviations (*n* = 6): *p < 0.05, **p < 0.01, ***p < 0.001 (*t*‐test).

To further demonstrate the enhanced photocatalytic eradication of MRSA biofilms and efficient osseointegration, we built an in vivo implantation model in rats to evaluate the anti‐biofilm abilities of the nanofilm under 808 nm NIRL irradiation. To minimize the number of animals used, pure Ti (the control group) and Ti‐CTO/RP were chosen for the animal tests. The treatment schedule is shown in **Figure** **6**A. After being immersed in MRSA suspension for 1 h, the samples were surgically implanted in the marrow cavities of rats’ femurs. After the biofilms were formed on the surfaces of the implants (three days postoperation), the surgical positions were irradiated by 808 nm NIRL. The implants had been heated up because of the high penetration power of NIRL. As Figure 6B shows, the change of temperature in situ was recorded using a thermal imager. The corresponding temperature curves indicated that the pure Ti group only reached ≈44 °C under 808 nm NIRL (Figure 6C). By contrast, the Ti‐CTO/RP group reached more than 50 °C within 2 min of irradiation and then maintained a temperature of ≈55 °C for 20 min. The results were consistent with the in vitro photothermal tests. Additionally, the rats irradiated by 808 nm NIRL three days postoperation were sacrificed and the implants were immediately extracted from bony tissues in order to observe the biofilms formation using SEM. As shown in Figure 6D, the viable MRSA biofilms were clearly observed on the surface of the pure Ti. Bacterial exocrine polymers (green arrows) were found adjacent to the biofilms, which was an important indicator of the binding of bacteria to each other in the gathering phase during the formation of the biofilms. Highly organized structures consisting of piles of colonies resembling mushrooms were also observed (blue arrows), which are typical symbols of mature biofilms after the gathering phase.^[^ ^38^ ^]^ However, the biofilms were severely damaged by the CTO/RP heterostructure after irradiation under 808 nm NIRL for 20 min. The biofilms were severely deformed as indicated by the red arrows. To further evaluate the long‐term effectiveness after the elimination of MRSA biofilms, the implants were collected and rolled in several laps on the LB agar plates to evaluate the remaining colony after seven days. The pure Ti group formed numerous MRSA bacterial colonies, as shown in Figure 6E. The collected pure Ti rods were also immersed in sterile LB broth at 37 °C for 24 h, and the LB broth became obviously turbid because of the growth of biofilms remaining on the surfaces of the Ti rods, suggesting that the immune system of rats is unable to eliminate MRSA biofilms. By contrast, only several bacterial colonies were observed in the Ti‐CTO/RP group and the LB broth was transparent. The results demonstrated that the Ti‐CTO/RP exhibited excellent anti‐biofilm ability with long‐term effectiveness under 808 nm NIRL. The anti‐biofilm efficiency was calculated based on the number of bacterial colonies. We found that 99.42% ± 0.22% of MRSA biofilms were eliminated in vivo by the Ti‐CTO/RP after irradiation under 808 nm NIRL (Figure 6F). Moreover, the bony tissues were harvested and stained with hematoxylin and eosin (H&E) and Giemsa in order to evaluate the condition of tissue inflammation. A number of neutrophils were found in the infected site along with the blood circulation, which was an indication of immune response to bacterial infections. A large number of lobulated neutrophils (black arrows) in the bone tissue adjacent to the implants were observed in the pure Ti group (Figure S19, Supporting Information), indicating severe infections and inflammation. However, only a few neutrophils were detected in the Ti‐CTO/RP group, suggesting a mild infections and effective biofilms elimination in the Ti‐CTO/RP group in vivo. In addition, bacterial residue in the bone tissue near the implants was also examined by Giemsa staining (Figure S20, Supporting Information). A load of MRSA (red arrows) were observed in the pure Ti group, whereas the number of detectable MRSA in the Ti‐CTO/RP group was minimal.

**Figure 7:**
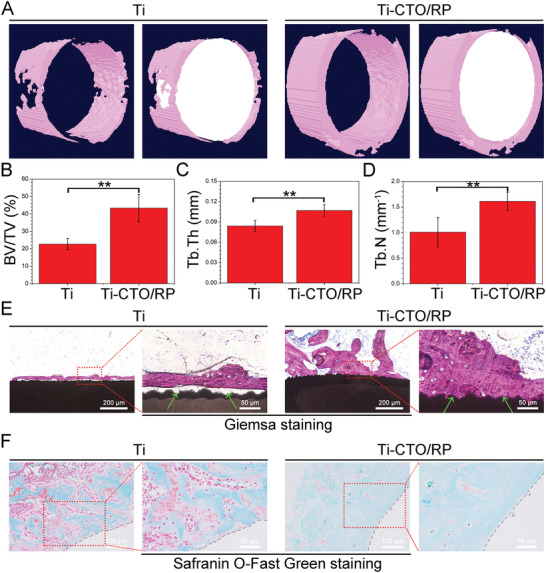
CTO/RP nanofilm for in vivo osteogenesis and bone‐implant osseointegration. A) The µ‐CT reconstructed 3D images of newly formed bones around the implants in the rats’ femurs after implantation for 28 days. The implants and the newly formed bones were marked in white and pink, respectively. B–D) The corresponding quantitative analysis results of (B) the bone volume fraction (BV/TV), (C) the trabecular thickness (Tb.Th), and (D) trabecular number (Tb.N). E) The evaluation of the mineralized bone tissue and osseointegration at the bone‐implant interfaces after stained with Giemsa. The ash black and amaranth indicated the implants and the mineralized bone tissue, respectively. Green arrows: bone‐implant interfaces. Scale bar in low magnification images (left): 200 µm. Scale bar in high magnification images (right): 50 µm. F) The evaluation of osteogenic differentiation at the bone‐implant interfaces after stained with Safranin‐O and Fast Green. Red color and green color indicated the chondrogenic differentiation and osteogenic differentiation, respectively. Black dashed lines: bone‐implant interfaces. Scale bar in low magnification images (left): 100 µm. Scale bar in high magnification images (right): 50 µm. Error bars indicate means ± standard deviations (*n* = 6): *p < 0.05, **p < 0.01, ***p < 0.001 (*t*‐test).

At the endpoint of four weeks, the rats were euthanized and the bony tissues were collected in order to evaluate the osteogenesis and bone‐implant osseointegration of different samples. The newly formed bony tissues were measured by a micro‐computed tomography (µ‐CT) system. As shown in **Figure** **7A**, the implants and the newly formed bones were marked in white and pink, respectively, in the reconstructed µ‐CT images. The bone volume around the surface of implant in the Ti‐CTO/RP group was visually larger than that of in pure Ti group. And the quantitative analysis of the newly formed bones was performed by a CTAn program. The bone volume fraction (bone volume/total volume, BV/TV) was first evaluated as indicated in Figure 7B. The BV/TV in Ti‐CTO/RP group was 43.39% ± 7.77%, which was almost twice as high as 22.69% ± 3.21% obtained in pure Ti group. The same observation was documented in the evaluation of both trabecular thickness (Tb.Th, Figure 7C) and trabecular number (Tb.N, Figure 7D). Consequently, Ti‐CTO/RP promoted the new bone formation around the implant after the elimination of MRSA biofilms.

Additionally, the harvested bones were further orderly processed by standard hard tissue and Giemsa staining to evaluate the mineralized bone tissue and osseointegration at the bone‐implant interfaces. And the corresponding histological photographs were collected by an optical microscope as indicated in Figure 7E. The ash black meant the implants while the amaranth indicated mineralized bone tissue. Only a small amount of bone tissue was observed on the surface of pure Ti after four weeks of implantation. Moreover, the bone‐implant interface was filled with fibrous connective tissues and bone‐implant contact was hardly observed (green arrows). By contrast, more mineralized deposits were detected on the surface of Ti‐CTO/RP and the newly generated bone tissue was closely bonded with the implant, indicating that Ti‐CTO/RP enhanced the osteogenesis and osseointegration. The collected bones were also stained with Safranin‐O and Fast Green to evaluate chondrogenic or osteogenic differentiation of different samples. Red meant the chondrogenic differentiation while green indicated osteogenic differentiation. As shown in Figure 7F, there were a lot of red areas in the pure Ti group, suggesting that the pure Ti group promoted chondrogenic differentiation. However, histological photograph was filled with green in Ti‐CTO/RP group, further demonstrating the enhancement of osteogenic differentiation.

Finally, the main organs of the treated rats were harvested and stained with H&E for biological safety assessment of the nanofilm. The internal organs (heart, liver, spleen, lung, and kidney) were not damaged in either the pure Ti group or the Ti‐CTO/RP group (Figure S21, Supporting Information), suggesting that the phototherapeutic heterostructures demonstrated excellent biological safety.

## 3 Conclusion

In summary, we constructed a novel perovskite‐based P–N heterojunction nanofilm through the combination of CTO and RP on a Ti plate. The formation of an internal electric field and the band offset effectively accelerated the separation and transfer of photogenerated charges under NIRL irradiation. Additionally, the rapid charge transfer induced DLDs, hot carrier relaxation and electron loops to achieve excellent energy conversion from optical energy to heat. Consequently, the nanofilm exhibited excellent NIRL‐driven photocatalytic and photothermal performances, which enable it to effectively eradicate mature MRSA biofilm infections on implants in vivo within a short time through the synergistic action of ROS and hyperthermia. Moreover, the rats’ MRSA biofilm infected orthopedic implant model indicated that the calcium and phosphorus sources induced superior biocompatibility and osteoconduction enable the subsequent bone regeneration and osseointegration after the eradication of MRSA biofilms. Therefore, this platform provides an advanced and ideal strategy for designing the safe and multifunctional surface on implants to address the clinical challenges.

## 4 Experimental Section

Experimental details can be found in the Supporting Information.

## Conflict of Interest

The authors declare no conflict of interest.

## Supporting information

Supporting Information

## Data Availability

The data that support the findings of this study are available from the corresponding author upon reasonable request.
