# Supplementary material for: Enhanced Near‐Infrared Photocatalytic Eradication of MRSA Biofilms and Osseointegration Using Oxide Perovskite‐Based P–N Heterojunction
Source: Adv Sci (Weinh). 2021 Jun 19;8(15):2002211. doi: 10.1002/advs.202002211 (PMC8336500; doi:10.1002/advs.202002211)
Supplement: Supplementary file 1 — Supporting Information [file ADVS-8-2002211-s001.pdf]

WILEY-VCH

© 2021 Wiley-VCH GmbH

ADVANCED SCIENCE
Open Access

Supporting Information

for *Adv. Sci.,* DOI: 10.1002/advs.202002211

Enhanced Near-Infrared Photocatalytic Eradication of MRSA Biofilms and Osseointegration Using Oxide Perovskite-Based P-N Heterojunction

Congyang Mao, Weidong Zhu, Yiming Xiang, Yizhou Zhu, Jie Shen, Xiangmei Liu, Shuilin Wu*, Kenneth M.C. Cheung, Kelvin Wai Kwok Yeung**

Supporting Information

Enhanced Near-Infrared Photocatalytic Eradication of MRSA Biofilms and Osseointegration Using Oxide Perovskite-Based P-N Heterojunction

Congyang Mao, Weidong Zhu, Yiming Xiang, Yizhou Zhu, Jie Shen, Xiangmei Liu, Shuilin Wu*, Kenneth M.C. Cheung, Kelvin Wai Kwok Yeung**

Experimental Section

Materials: Medical-grade pure titanium (Ti) rods (ϕ2×6 mm) and pure Ti plates (ϕ6×2 mm and ϕ22×2 mm) were obtained from Fu-Tai Metal Materials Co. (China). Calcium oxide (CaO, 99.95%, metals basis, -20 Mesh Powder) was purchased from Alfa Aesar (China). Amorphous red phosphorus (RP) was obtained from Kermel (China). Ethanol (≥ 99.7%) was obtained from the Sinopharm Chemical Reagent Co., Ltd (China). The 2,2,6,6-tetramethylpiperidine (TEMP), titanium butoxide (≥ 99.0%), 1, 3-Diphenylisobenzofuran (DPBF) and thiazolyl blue tetrazolium bromide (MTT) were obtained from Aladdin (China). The 5,5-dimethyl-1-pyrroline-N-oxide (DMPO) was obtained from Sigma-Aldrich (America). The BCA Protein Assay Kit (Cat. # PC0020) was obtained from Solarbio Life Science (China). The microplate test kit (alkaline phosphatase assay kit) was obtained from the Nanjing Jiancheng Bioengineering Institute (China). The Reactive Oxygen Species Assay Kit (Cat. # S0033) was purchased from Beyotime Biotechnology (China). Live & Dead Bacterial Staining Kit (Cat. # 40274ES60), Tetramethylrhodamine isothiocyanate (TRITC)-conjugated phalloidin (Cat. # 40734ES75), and 4',6-diamidino-2-phenylindole (DAPI) stain solution (Cat. # 40728ES03) were purchased from Yeasen (China). The Total RNA Kit I (50) (Cat. # R6834-01) was obtained from Omega Bio-tek (America). The PrimeScript RT Master Mix (Cat. # RR036A) and TB Green Premix Ex Taq II (Cat. # RR820A) were purchased from TaKaRa (Japan).

Preparation of red phosphorus powder: First, 6 grams of amorphous RP was mixed with 50 mL of deionised water and then treated by the hydrothermal method at 200 ^{o}C for 12 h. After the hydrothermal reaction was finished, the crude product was washed several times with deionised water and further purified by a second hydrothermal treatment under the same conditions to thoroughly remove the oxide layer on the surface of the RP. The RP was again washed several times with deionised water and dried in a vacuum oven at 60 ^{o}C for 12 h. Finally, the dried sample was ground into a homogeneous powder in a mortar and stored in a nitrogen (N_2) atmosphere for subsequent experiments.

Fabrication of heterostructure coating: The pure Ti plates were first mechanically polished with silicon carbide (SiC) paper with grain sizes of 240, 400 and 800 sequential. The polished samples were then ultrasonically washed with acetone, ethanol and deionised water sequentially to obtain the pure Ti group.

The treated Ti plates were then placed in a 100-mL Teflon reactor. Next, 0.5 g CaO was dissolved in 50 mL of deionised water by heat treatment and then moved into the reactor. Subsequently, the mixture of 2.5 mL of titanium butoxide and 7.5 mL of ethanol was added into the above solution. After that, 10 ml of deionised water was added and thermally treated at 150 ^{o}C for 24 h. The thermally treated samples were washed repeatedly with deionised water and finally put in a muffle furnace at 650 ^{o}C for 1 h at a heating rate of 10 ^{o}C min^{-1} to obtain a coating of calcium titanate ($CaTiO_3$), which was labelled Ti-CTO.

For the RP coating, 0.3 grams of RP powder was first uniformly placed into a porcelain boat. The pure Ti and Ti-CTO samples were then placed on top of the RP powder and moved into a chemical vapour deposition (CVD) tube furnace. After the tube furnace was vacuumed, the temperature was increased to 650 ^{o}C at a heating rate of 10 ^{o}C min^{-1} and kept at 650 ^{o}C for 1 h. The temperature was subsequently reduced to 280 ^{o}C at a cooling rate of 10 ^{o}C min^{-1} and kept at 280 ^{o}C

for 10 h. Finally, the tube was naturally cold to obtain the fibre-phase RP film on the surface of the Ti and Ti-CTO, which were labelled Ti-RP and Ti-CTO/RP, respectively.

Characterisation of different samples: A field-emission scanning electron microscope (FESEM, ZEISS Sigma 500) was used to observe the morphologies of different samples. The biofilms were observed with an SEM (JSM6510LV). The detailed structures of the samples were observed with a transmission electron microscope (TEM, FEI Tecnai G2 20 S-TWIN). A Nano Indenter (Hysitron TI-Premier) equipped with a Berkovich pyramidal-shaped diamond tip was used to evaluate the adhesion strength of coating. A UV-Vis-NIR spectrometer (UV-Vis-NIR, UV-3600, Shimadzu) was used to obtain the UV-Vis-NIR absorption spectra of different coatings. A photothermal imager (Testo 875i) was used to collect the photothermal images and record the temperatures. An electrochemical workstation (CHI660E) was used to test the photocurrent densities and electrochemical impedance spectroscopy (EIS) of different samples. A fluorescence spectrophotometer (Perkin Elmer LS55) was used for photoluminescence (PL) spectra. A micro-computed tomography (μ-CT) system (Skyscan 1176, Bruker) was used to scan the rats for quantitative analysis of the new bone generation.

Photocatalytic and photothermal tests: ROS generation was first detected using the Reactive Oxygen Species Assay Kit. According to the manufacturer's instructions, after hydrolysing the DA molecule from 2',7'-dichlorodihydrofluorescein diacetate (DCFH-DA) by sodium hydroxide, the samples (ϕ6×2 mm) were placed into 96-well plates with 150 μL of DCFH solution and then discontinuously irradiated with 808 nm NIRL (0.5 W cm^{-2}) for 20 min. The level of ROS generation at 2-min intervals was evaluated with a microplate reader (SpectraMax i3, Molecular Devices) with excitation at 488 nm and emission at 525 nm.

An ESR spectrometer (JES-FA200) was then used to identify the species of ROS, and 2,2,6,6-tetramethylpiperidine (TEMP, 50 mM) was used to detect singlet oxygen ($^1O_2$), whereas 5,5-dimethyl-1-pyrroline-N-oxide (DMPO, 0.1 M) was used to trap hydroxyl radicals (•OH). The

samples were briefly placed into 150 μL of spin-trapping solution in 96-well plates and then irradiated with 808 nm NIRL (0.5 W cm^{-2}) for 20 min. The ESR spectra were obtained with an ESR spectrometer at a micro-frequency of 8.93 GHz and micro-power of 3 mW.

DPBF was used to further detect $^1O_2$. In brief, DPBF was dissolved in N,N-Dimethylformamide (DMF) to obtain the working solution (50 μg mL^{-1}). The samples were placed into 150 μL of above solution in 96-well plates and then irradiated with 808 nm NIRL (0.5 W cm^{-2}) for 20 min, and then 100 μL of irradiated solution was taken to test the absorption spectra from 350-500 nm. The pure DPBF working solution was used as control.

In the photothermal tests, the samples (ϕ6×2 mm) were placed in 96-well plates with 150 μL of phosphate buffer saline (PBS) solution and then continuously irradiated with 808 nm NIRL (0.5 W cm^{-2}) for 20 min. A photothermal imager (Testo 875i, Germany) was used to collect the photothermal images and record temperatures. Moreover, the suspension temperature variations above the Ti-CTO/RP were recorded under 808 nm NIRL irradiation for 20 min, then the laser was turned off and the Ti-CTO/RP was naturally cooled for another 20 min for a total of five cycles of laser on/off to evaluate the photothermal stability of the sample.

Theoretical calculations: The CP2K package was used to perform all the hybrid DFT calculations, and the system was described using the Perdew-Burke-Ernzerhof (PBE) functional with the Grimme D3 correction method. Using the framework of the Gaussian and plane waves method, the Kohn-Sham DFT was used as the electronic structure method. The molecules were described using the Goedecker-Teter-Hutter (GTH) pseudopotentials and DZVP-MOLOPT-GTH basis sets. A plane-wave energy cut-off of 500 Ry was also employed. Importantly, the electronic structures of the CTO, RP and CTO/RP heterojunctions were corrected using the Heyd-Scuseria-Ernzerhof (HSE06) hybrid functional model.

The work function was defined as follows (Equation (S1)):

$\Phi = E_{vac} - E_F$ (S1)

where E_{vac} was the energy of the electrostatic potential in the vacuum and the vacuum was set at 0. E_F was the Fermi level of the system.

The VB and CB edge potentials were defined as follows (Equations (S2) and (S3), respectively):

$E_{VB} = \chi - E_e + 0.5E_g$ (S2)

$E_{CB} = E_{VB} - E_g$ (S3)

where χ values were the absolute electronegativity and the χ values of CTO and RP were 5.04 and 5.62 eV, respectively. E_e (4.5 eV) was the free electronic energy on the hydrogen scale. E_g was the energy of the band gap.

The charge density difference was defined as follows (Equation (S4)):

$\Delta\rho = \rho(\text{CTO/RP}) - \rho(\text{CTO}) - \rho(\text{RP})$ (S4)

where the $\Delta\rho$ was the charge difference between ρ(CTO) and ρ(RP). The ρ(CTO/RP) was the total density.

In vitro antibacterial activity assay: *E. coli* and MRSA with the concentration of 10^7 CFU mL^{-1} were used to evaluate the bacterial killing abilities of different samples. The bacterial suspension was first diluted 100-fold by LB broth. The samples (ϕ6×2 mm) in triplicate were placed in 96-well plates before 150 μL diluted bacterial suspension was moved into 96-well plates. The 96-well plates containing samples and bacterial suspension was irradiated with or without the 808 nm NIRL (0.5 W cm^{-2}) for 20 min, and the pure Ti group in the darkness was served as control group. And the irradiated bacterial suspension was further diluted 100-fold by LB broth and 20 μL diluted bacterial suspension was then collected and spread on LB agar plate. Finally, the viable colony units were

formed on LB agar plate after 24 h for *E. coli* and 48 h for MRSA at 37 ^{o}C. And the CFU were obtained by counting the bacterial colonies from irradiated bacterial suspension treated by different samples. And the corresponding antibacterial ratio was calculated as follows Eq. (S5).

$$\text{Antibacterial ratio (\%)} = (CFU_c - CFU_e) * 100/CFU_c \qquad (S5)$$

Where CFU_c is the average CFU in the control group, and CFUe is the average CFU in the experimental groups.

And the samples were collected and the morphologies of bacteria on the surface of samples were further evaluated by SEM and TEM observation. The samples were rinsed repeatedly with PBS (pH=7.4). The bacteria on the surface of samples were then fixed for 2 h with 2.5% glutaraldehyde before dehydrated in graded ethanol (30, 50, 70, 90 and 100%) for 15 min. Finally, the dehydrated samples were air-dried overnight for tests. To examine the morphology of MRSA by TEM technique, the irradiated MRSA bacteria were centrifuged and then fixed by glutaraldehyde and OsO_4 orderly. A gradient of ethanol (30, 50, 70, 90 and 100%) was applied to dehydrate the MRSA bacteria. The samples were subsequently placed into Epon before embedded with Epon/Araldite resin. Lastly, the fixtures of MRSA bacteria were sectioned for TEM examination.

For the MRSA biofilm eradication test, a 200 μL of MRSA suspension (10^7 CFU mL^{-1}) was added to the 96-well plates containing Ti and Ti-CTO/RP and subsequently cultured at 37 ^{o}C for 3 d. The culture medium was changed every 12 h. Afterward, the samples were irradiated with 808 nm NIRL (0.5 W cm^{-2}) for 20 min. The *in vitro* biofilm eradication properties of the samples were evaluated by SEM technique and live/dead staining (Live & Dead Bacterial Staining Kit) according to the manufacturer's instructions.

Cell culture: Rat' MSCs were obtained from Tongji hospital in Wuhan, China. The MSCs were cultured in 85% DME/F-12 1:1 (1X) medium (HyClone, contained 2.50 mM L-Glutamine and 15 mM HEPES Buffer) and supplemented 15% fetal bovine serum (FBS) in an incubator of 5%

CO_2 and humidified atmosphere at 37 ^oC. The MSCs at three passage were used in the subsequent cell tests.

In vitro cytotoxicity tests: The MSCs at the concentration of 10^5 cells mL^{-1} was used in Thiazolyl Blue Tetrazolium Bromide (MTT) evaluation. Briefly, the samples (ϕ6×2 mm) in triplicate were placed in 96-well plates and 200 μL MSCs were then seeded onto the samples, and pure Ti group was served as the control. The growth medium was refreshed every 2 days. After culturing for 1, 3 and 7 days, the growth medium was replaced by 200 μL MTT solutions (0.5 mg mL^{-1} in PBS) and incubated in an incubator for another 4 h to form formazan. Subsequently, the MTT solution was discarded and 200 μL dimethyl sulfoxide (DMSO) was used to dissolve formazan under continuous shaking for 15 min. Afterwards, 100 μL dissolved formazan was collected and measured at 490 nm on a microplate reader (SpectraMax i3, Molecular Devices). And the corresponding cell viability was calculated as follows Eq. (S6).

$$\text{Cell viability (\%)} = OD_e * 100/OD_c \qquad \text{(S6)}$$

Where OD_c is the average optical density (OD) value at 490 nm in the control group, and OD_e is the average OD value at 490 nm in the experimental groups.

For the cellular fluorescence tests, the samples (ϕ6×2 mm) were placed in 96-well plates before 200 μL MSCs (10^4 cells mL^{-1}) were seeded onto the samples. After culturing for 24 h, filamentous actin (F-actin) and nucleus of MSCs were stained by TRITC Phalloidin and DAPI stain solution, respectively, according to the manufacturer’s instructions. Finally, the samples were air-dried overnight and observed by inverted fluorescence microscope (IFM, IX73, Olympus).

Total intracellular protein extraction and ALP activity assays: The MSCs at the concentration of 10^5 cells mL^{-1} was used in ALP investigation. Briefly, the samples (ϕ6×2 mm) in triplicate were placed in 96-well plates and 200 μL MSCs were then seeded onto the samples, and pure Ti group was served as the control. The growth medium was refreshed every 2 days. After culturing for 3, 7

and 14 days, the growth medium was replaced by 100 μL 0.1% Triton X-100 to lyse MSCs at 37 ^{o}C for 1 h. Subsequently, the total intracellular protein extraction and alkaline phosphatase (ALP) were measured by BCA Protein Assay Kit and Alkaline Phosphatase Assay Kit, respectively, according to the manufacturer's instructions. Finally, the corresponding ALP activities of different samples was normalized to the total protein content.

Total RNA extraction and quantitative real-time polymerase chain reaction (qRT-PCR): The MSCs at the concentration of 10^5 cells mL^{-1} was used in qRT-PCR evaluation of different samples. Briefly, the samples (ϕ22×2 mm) in triplicate were placed in 12-well plates and 1 mL MSCs were then seeded onto the samples, and pure Ti group was served as the control. The growth medium was refreshed every 2 days. After culturing for 7 and 14 days, Total RNA Kit I (50) was used to extract RNA based on instructions and NanoDrop 2000 spectrophotometer (Thermo Scientific) was used to measure the concentration of extracted RNA. Afterward, cDNA was synthesized by reverse-transcription of 500 ng extracted RNA using PrimeScrip RT Master Mix based on instructions. Finally, the qRT-PCR was performed and quantified by TB Green Premix Ex Taq II with the CFX Connect™ Real-Time System (Bio-Rad) based on instructions. The relative expression of different genes was evaluated by the $2^{-\Delta\Delta Ct}$ method and normalized to the corresponding gene glyceraldehyde-3-phosphate dehydrogenase (GAPDH) values from different samples. The primer sequences of MSCs were as follows: glyceraldehyde-3phosphate dehydrogenase (GAPDH) forward: 5'- GCCTCGTCTCATAGACAAGATGGT -3'; GAPDH reverse: 5'-GAAGGCAGCCCTGGTAACC -3'; *ALP* forward: 5'-AGCGACACGGACAAGAAGC-3'; *ALP* reverse: 5'-GGCAAAGACCGCCACATC-3'; *COL I* forward: 5'-CCTGAGCCAGCAGATTGA-3'; *COL I* reverse: 5'-TCCGCTCTTCCAGTCAG-3'; *OCN* forward: 5'-AAGCCCAGCGACTCTGAGTCT-3'; *OCN* reverse: 5'-CCGGAGTCTATTCACCACCTTACT-3'; *OPN* forward: 5'-TCCTGTCTCCCGGTGAAAGT-3'; *OPN* reverse: 5'-GGCTACAGCATCTGAGTGTTTGC-3'; *OSX* forward: 5'-CGGCAAGGCTTCGCATCTG-3';

OSX reverse: 5’-GGAGCAGAGCAGACAGGTGAACT-3’; *Runx-2* forward: 5’-AATGCCTCCGCTGTTATG-3’; *Runx-2* reverse: 5’-TTCTGTCTGTGCCTTCTTG-3’.

In vivo implantation, anti-biofilm and osteogenesis experiments: The male Sprague Dawley rats (380-400 g) were purchased from Hubei Provincial Centers for Disease Prevention and Control, and animal experiments were approved by the Department of Orthopedics, Union Hospital, Tongji Medical College, Huazhong University of Science and Technology, Wuhan, China. All rats were kept and treated according to the Animal Management Rules of the Ministry of Health of the People’s Republic of China and the Guidelines for the Care and Use of Laboratory Animals of China. To save animals, forty-eight rats were randomly divided into two groups (pure Ti and Ti-CTO/RP) based on the *in vitro* results and individually raised in cages. Each group contained twenty-four rats, and twelve rats were used for anti-biofilm test while other twelve rats were used for osteogenesis and osseointegration evaluation. For the surgery, general anaesthesia was initiated by the intraperitoneal injection of pentobarbitone (40 mg kg^{-1} body weight), and then surgical antiseptic procedures, such as hair shaving and iodophor disinfectant, were carried out. Afterwards, perforations in the femur with diameters of 2 mm were locally generated using a dental drill. The pure Ti and Ti-CTO/RP rods ($\phi 2\times 6$ mm) were immersed in MRSA at a concentration of 10^7 CFU mL^{-1} for 1 h before implanted into the intramedullary canal. Subsequently, layer-by-layer closure was applied to suture the wound. After three days, the implanted sites were irradiated under 808 nm NIRL (0.8 W cm^{-2}) for 20 min after MRSA biofilms formed on the surfaces of implants. After the irradiation, six rats in each group were sacrificed and the implants were removed from the bones. The MRSA biofilms on the surfaces of the implants were fixed and dehydrated for SEM observation. After seven days, another six rats in each group were sacrificed. The implants were collected and rolled in several laps on the LB agar plates to form the colony units. Afterwards, the implants were immersed in sterile LB broth at 37 ^{o}C for 24 h to observe the growth of the residual MRSA on the surfaces of the implants. Next, the bones were harvested and stained with H&E and

Giemsa to evaluate inflammation. After 4 weeks, the residual six rats in each group were euthanized and the bones were resected. Half of bones in each group were stained with Safranin-O and Fast Green to evaluate chondrogenic or osteogenic differentiation. Another half was embedded for the standard hard tissue processing. After stained with Giemsa, the mineralized bone tissues around the implants were observed by an optical microscope. Finally, the major organs were also resected and stained with H&E for biological safety assessment.

Statistical Analysis: The data from all the experiments were analyzed by mean values ± standard deviations (SD) with $n \geq 3$. The statistical analysis of multiple comparisons was performed by using a one-way analysis of variance (ANOVA) program followed by Tukey's post hoc test. The statistical analysis between two groups was performed by utilizing a two-sample Student's *t*-test. In addition, P values of $^*p < 0.05$, $^{**}p < 0.01$, and $^{***}p < 0.001$ were considered as statistically significant.

Supplementary Figures

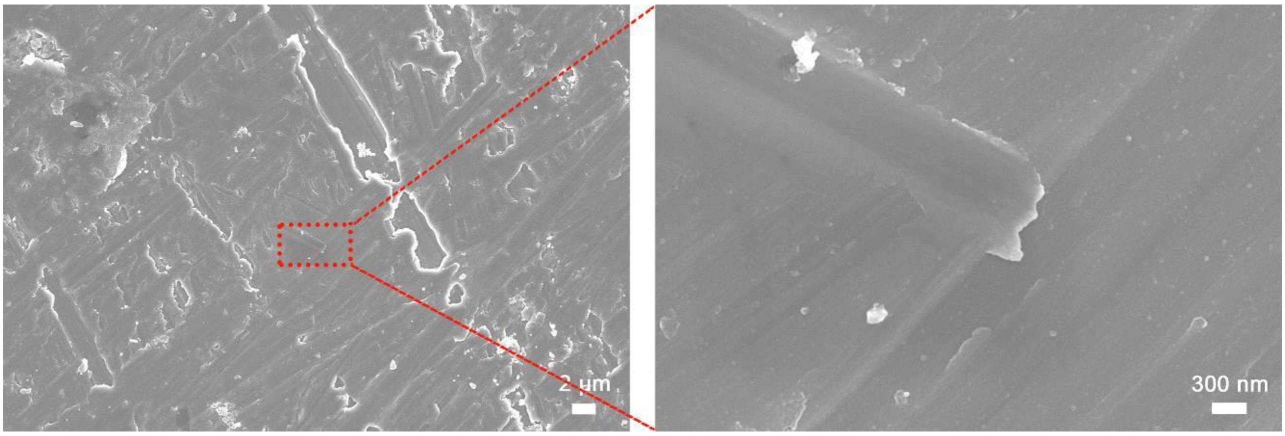

Figure S1. SEM images of pure Ti. Scale bar in low magnification images: 2 μm; Scale bar in high magnification images: 300 nm.

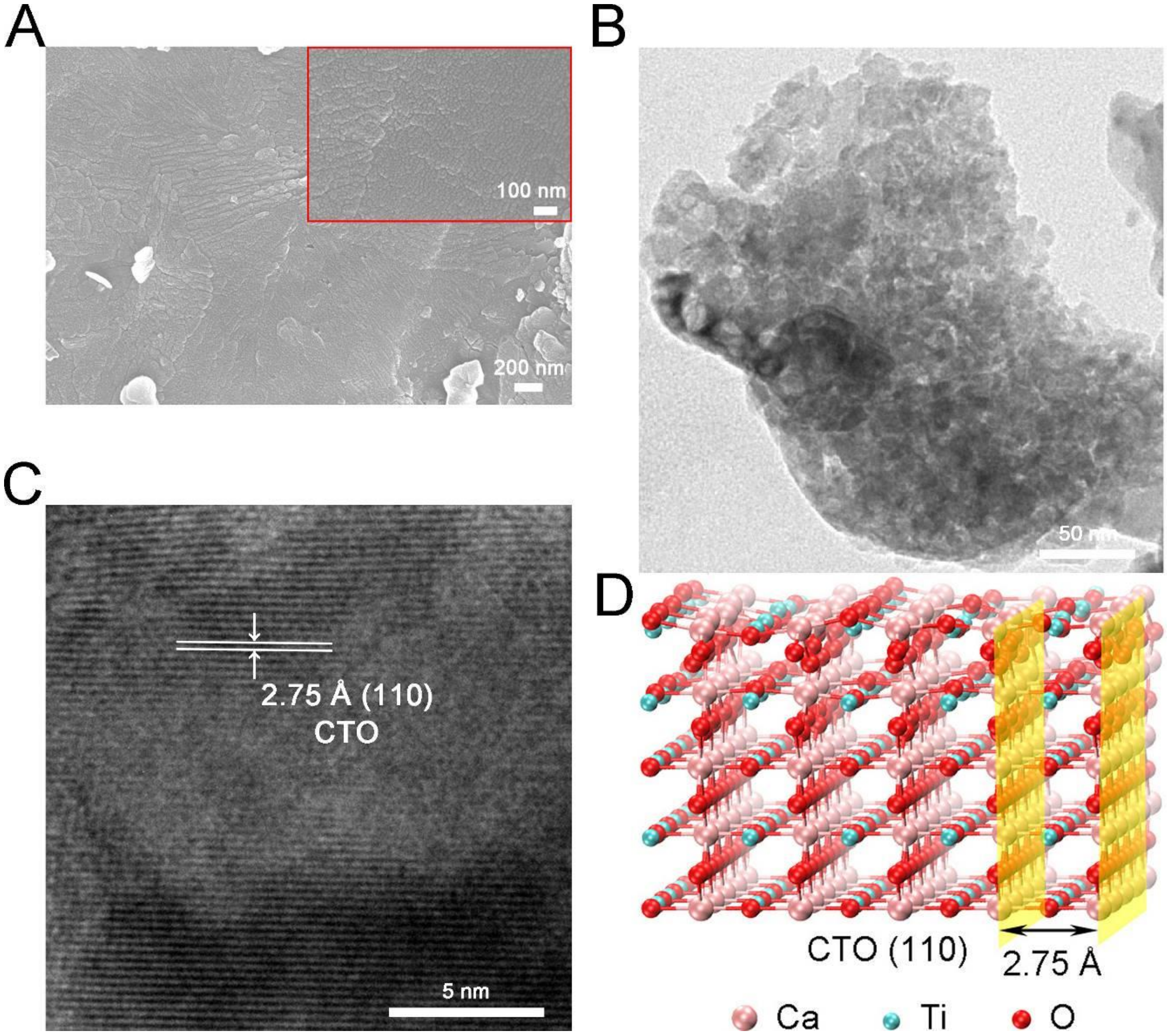

Figure S2. (A) SEM images of CTO nanofilm. Scale bar: 200 nm. The insert is the high magnification SEM images. Scale bar: 100 nm. (B) Transmission electron microscopy (TEM) image of CTO nanofilm. Scale bar: 50 nm. (C) HRTEM image of CTO nanofilm and corresponding lattice spacing calibration of CTO nanofilm. Scale bar: 5 nm. (D) The geometric structures of CTO, where pink, pale green, and red spheres represent Ca, Ti, and O atoms, respectively. The yellow plane refers to the corresponding lattice facets.

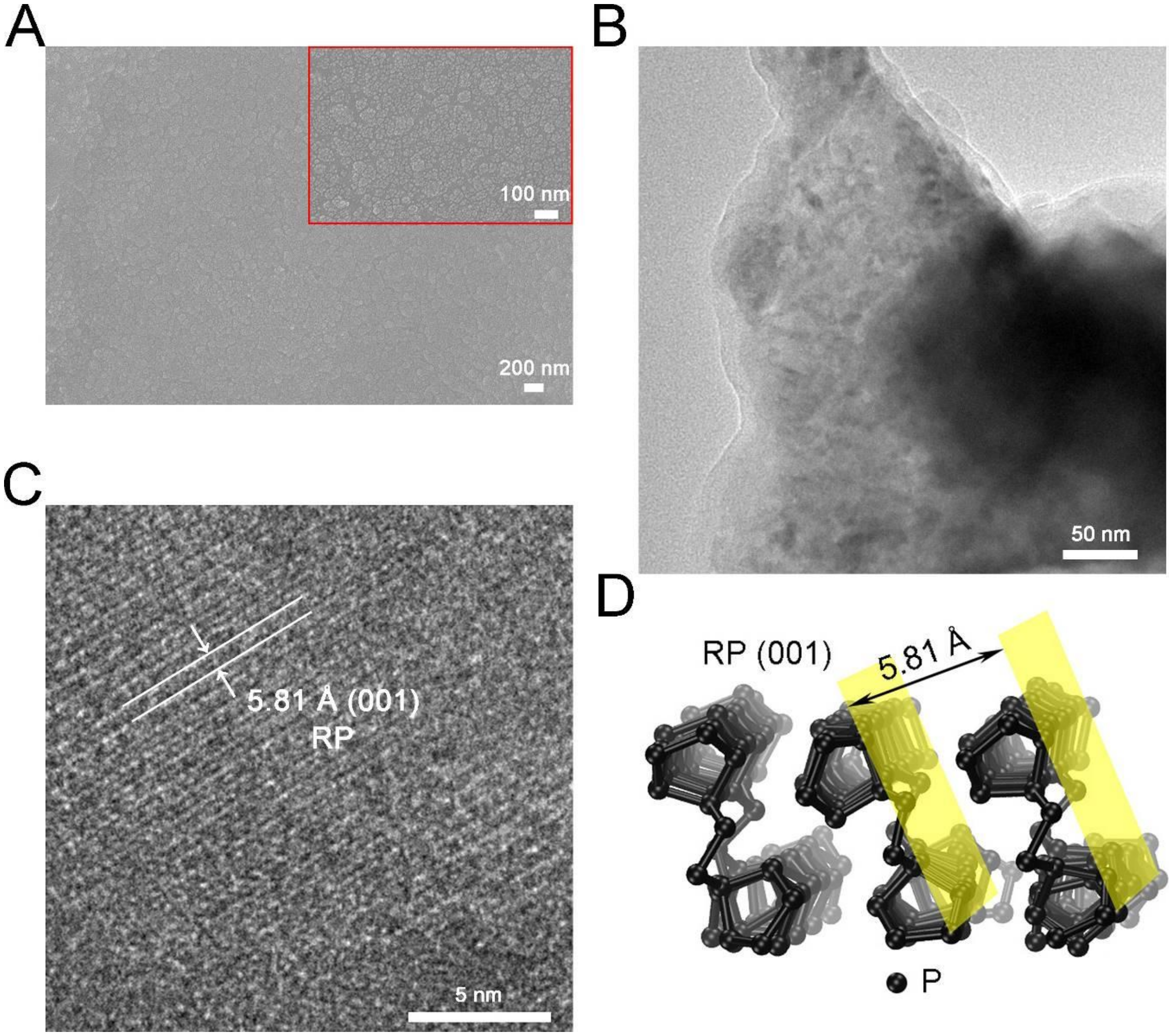

Figure S3. (A) SEM images of RP nanofilm. Scale bar: 200 nm. The insert is the high magnification SEM images. Scale bar: 100 nm. (B) TEM image of RP nanofilm. Scale bar: 50 nm. (C) HRTEM image of RP nanofilm and corresponding lattice spacing calibration of RP nanofilm. Scale bar: 5 nm. (D) The geometric structures of fibrous RP, where black spheres represent P atoms. The yellow plane refers to the corresponding lattice facets.

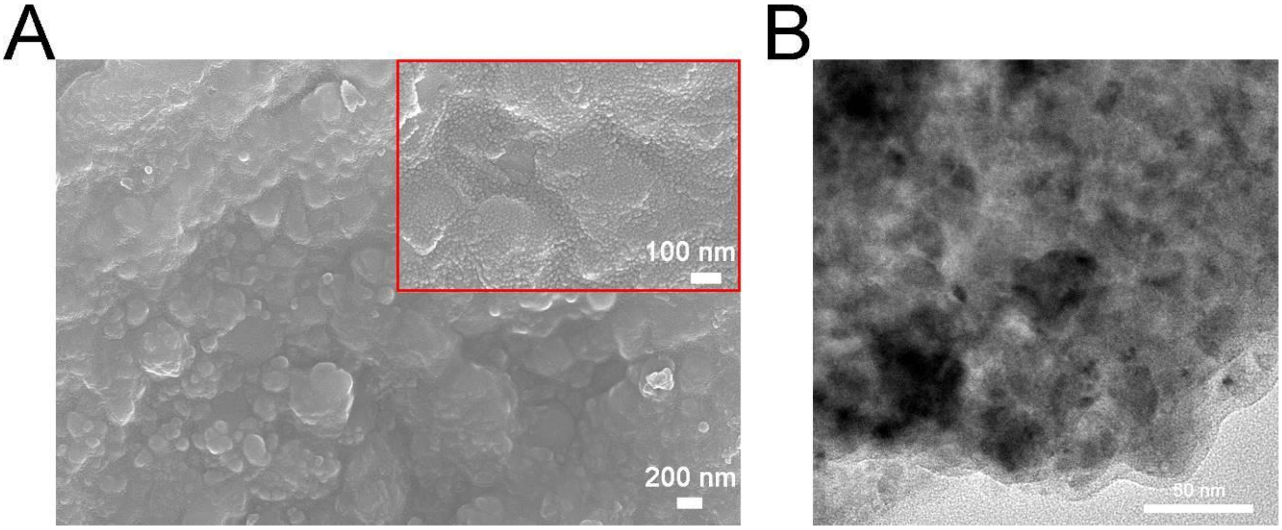

Figure S4. (A) SEM images of CTO/RP P-N heterojunction nanofilm. Scale bar: 200 nm. The insert is the high magnification SEM images. Scale bar: 100 nm. (B) TEM image of CTO/RP P-N heterojunction nanofilm. Scale bar: 50 nm.

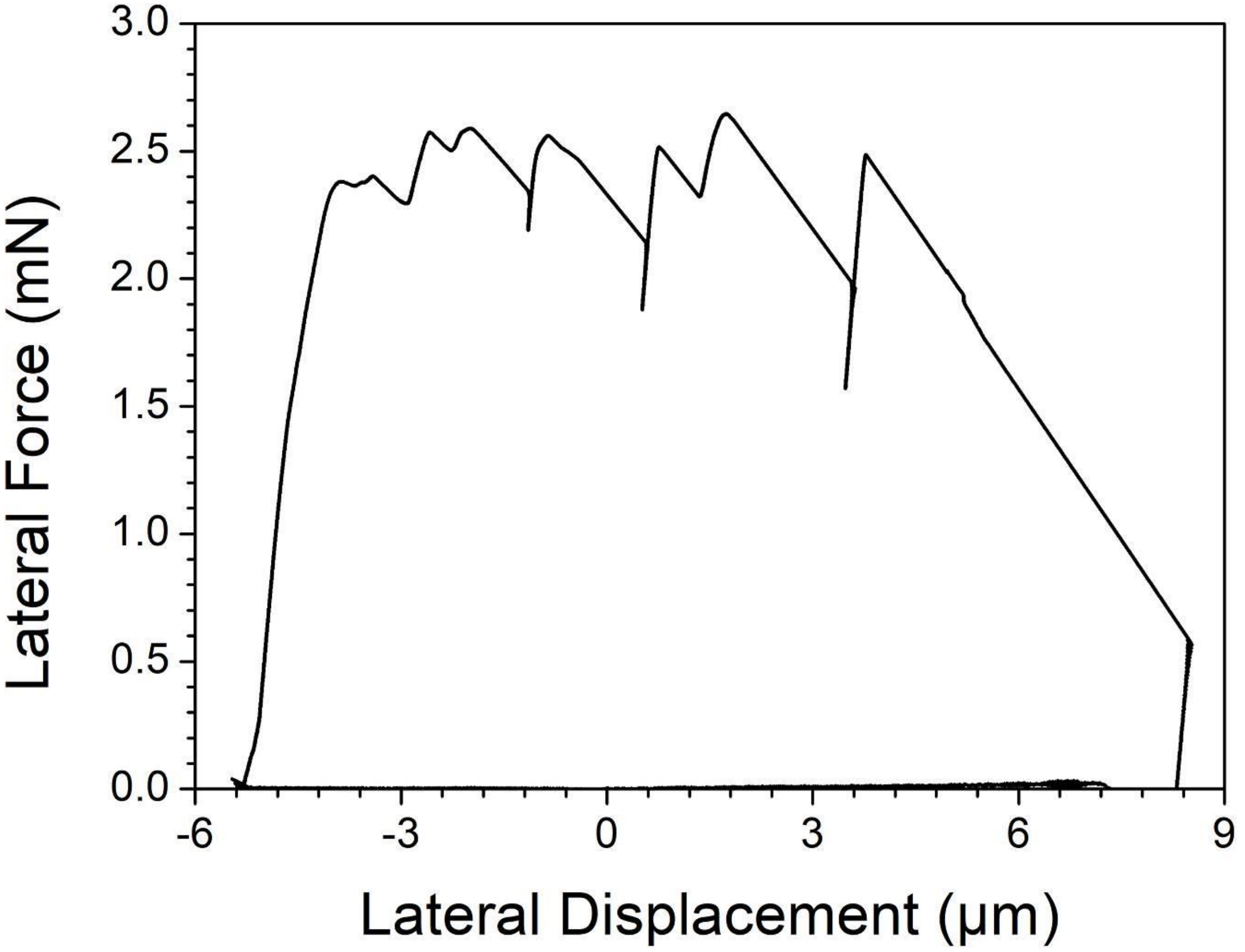

Figure S5. Lateral force-lateral displacement curve of CTO/RP coating acquired by the scratch test.

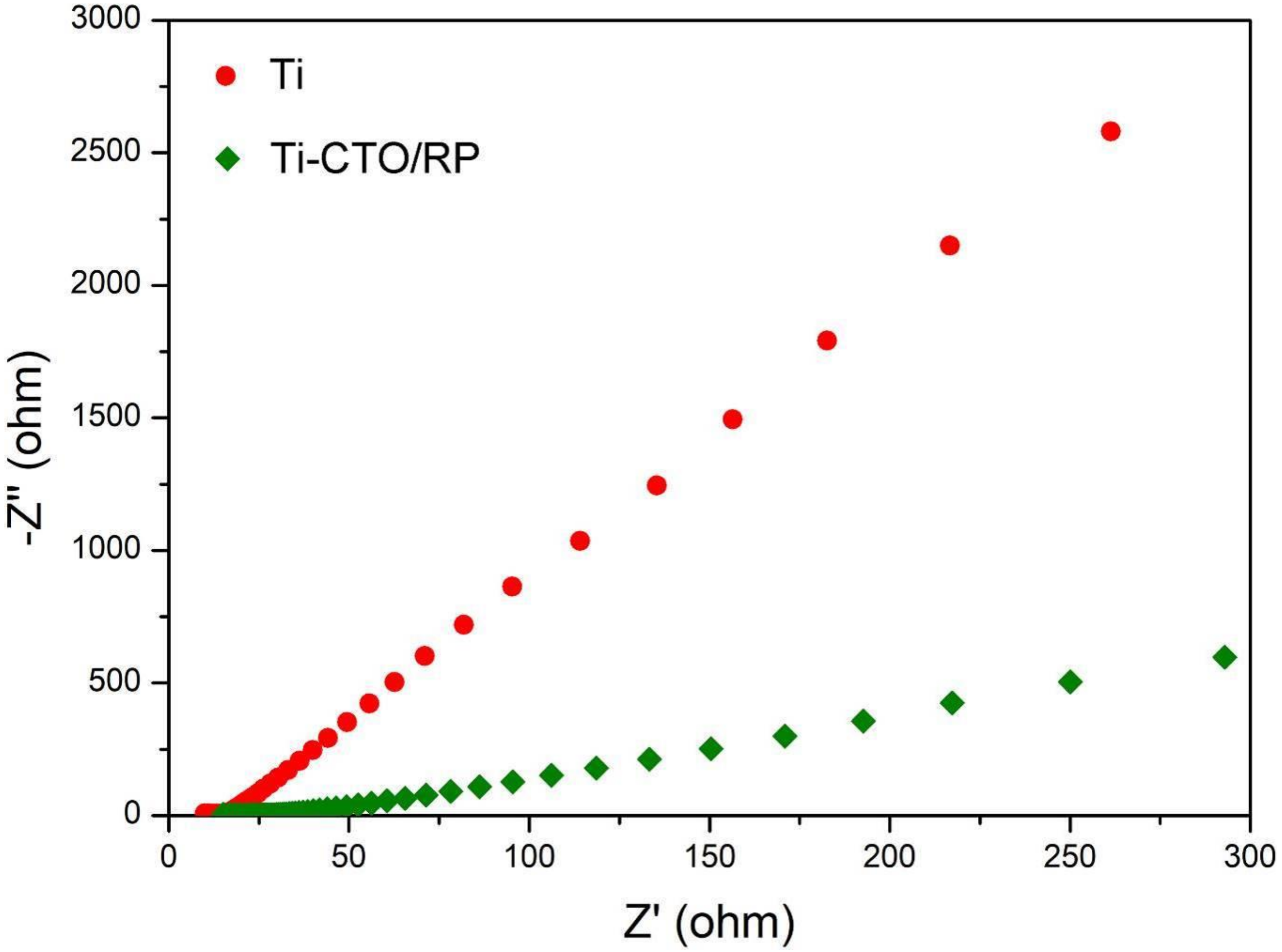

Figure S6. EIS of Ti and Ti-CTO/RP by electrochemical workstation.

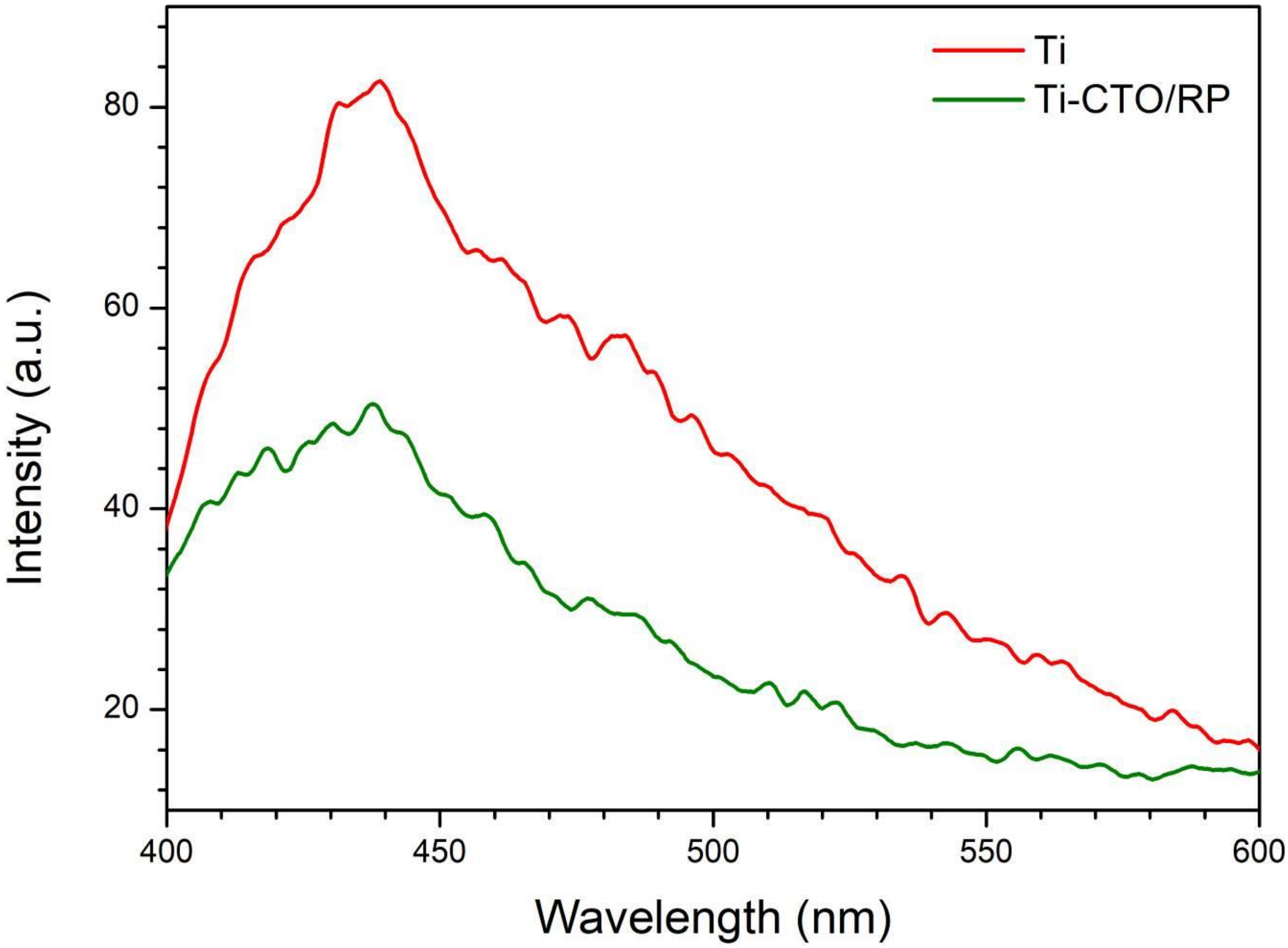

Figure S7. PL spectra of Ti and Ti-CTO/RP.

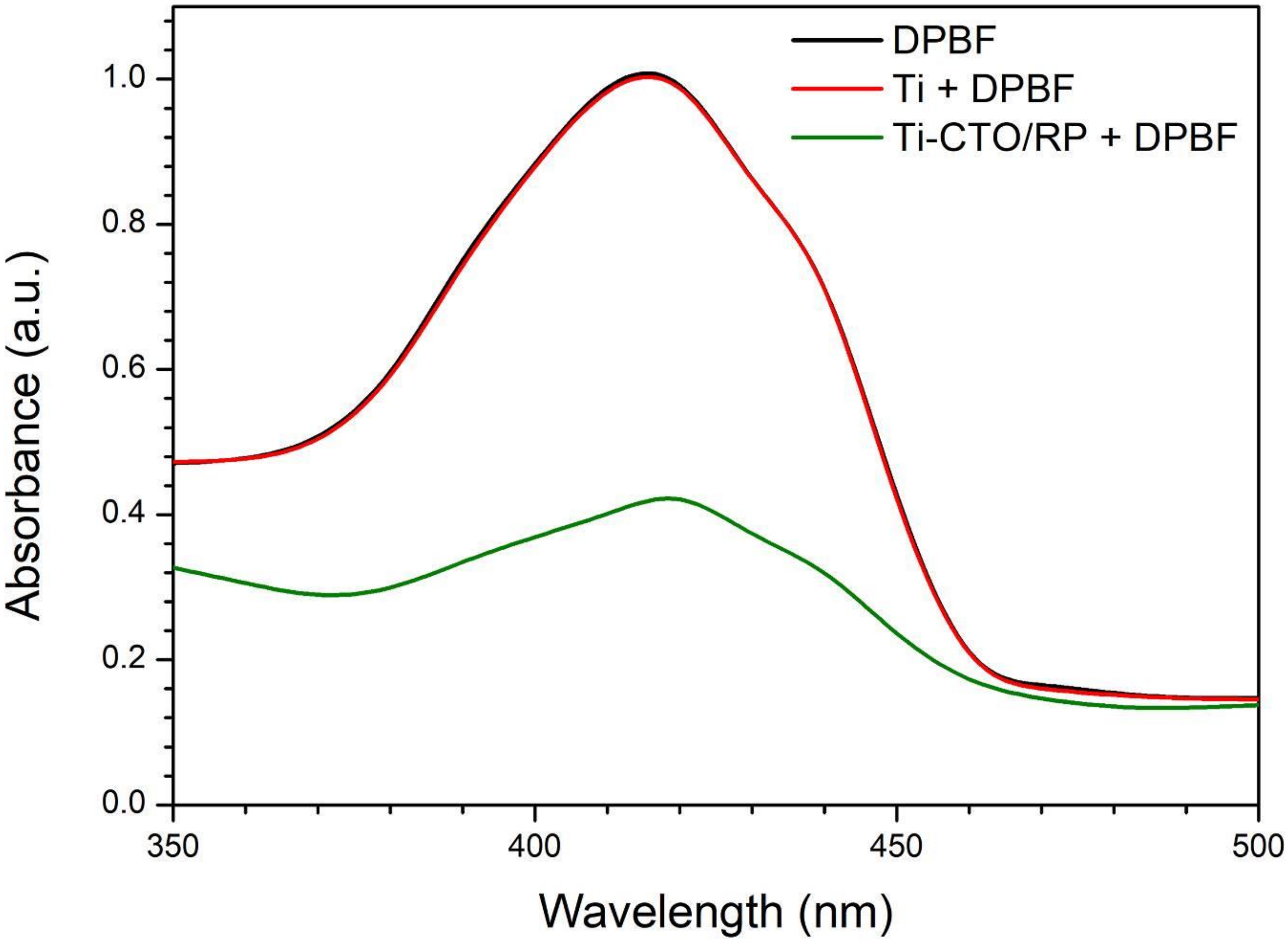

Figure S8. Absorption spectra of the DPBF in groups of DPBF, Ti and Ti-CTO/RP after irradiated with 808 nm NIRL (0.5 W cm^{-2}) for 20 min. The pure DPBF working solution was used as control.

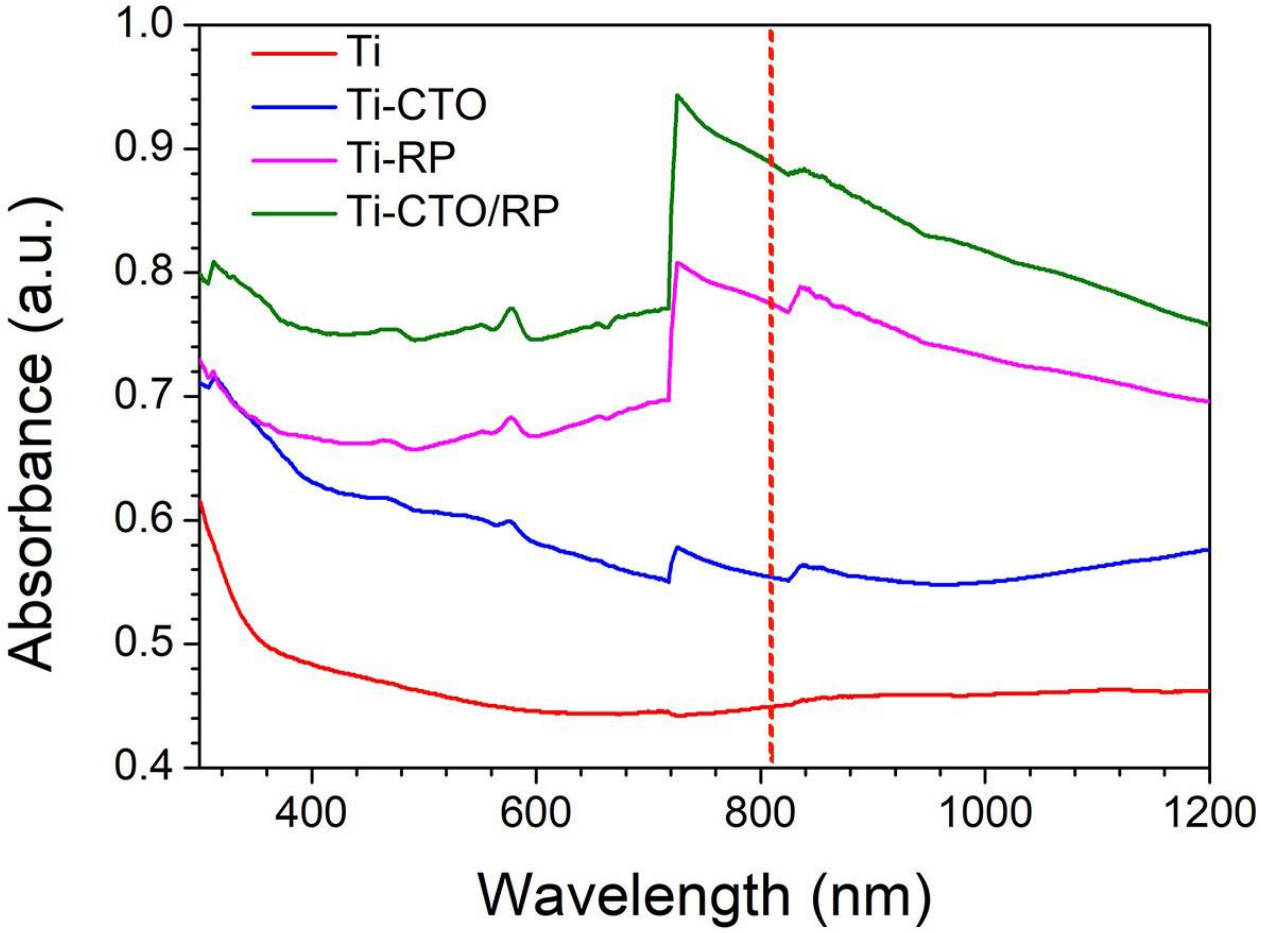

Figure S9. Visible-NIR absorption of Ti, CTO, RP and CTO/RP, where the red vertical dashed line indicates the absorption at the wavelength of 808 nm.

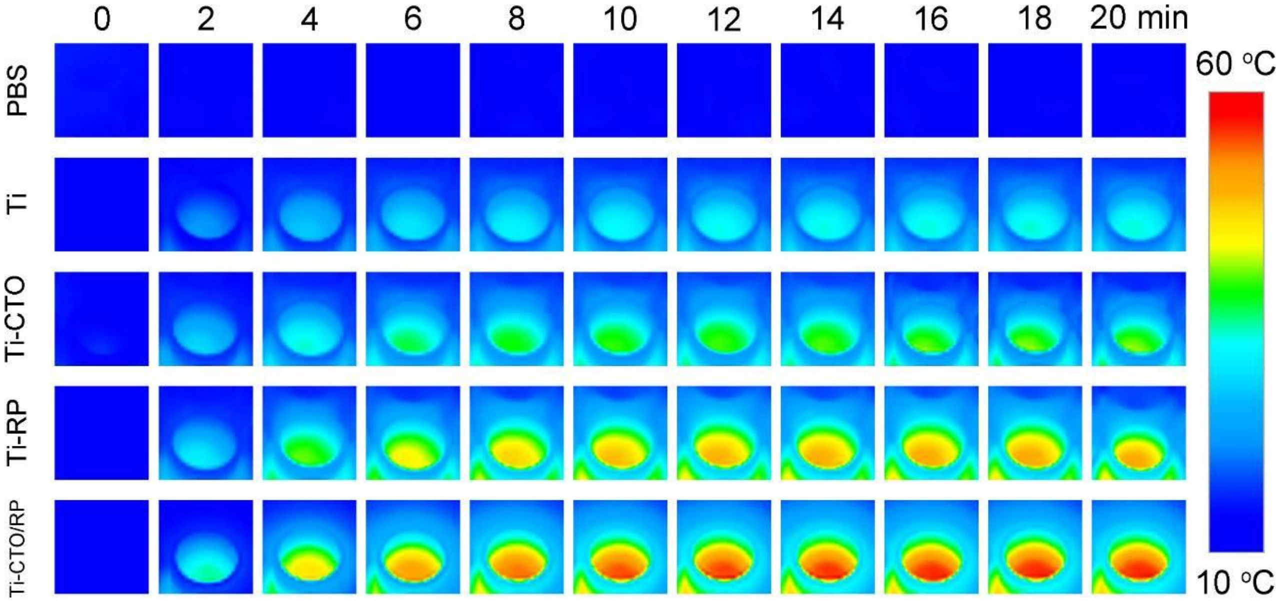

Figure S10. The thermal images versus time comparison of CTO/RP and the other samples show the best photothermal ability of CTO/RP under NIRL irradiation. The red and blue colors in the thermal images represent higher and lower temperature, respectively.

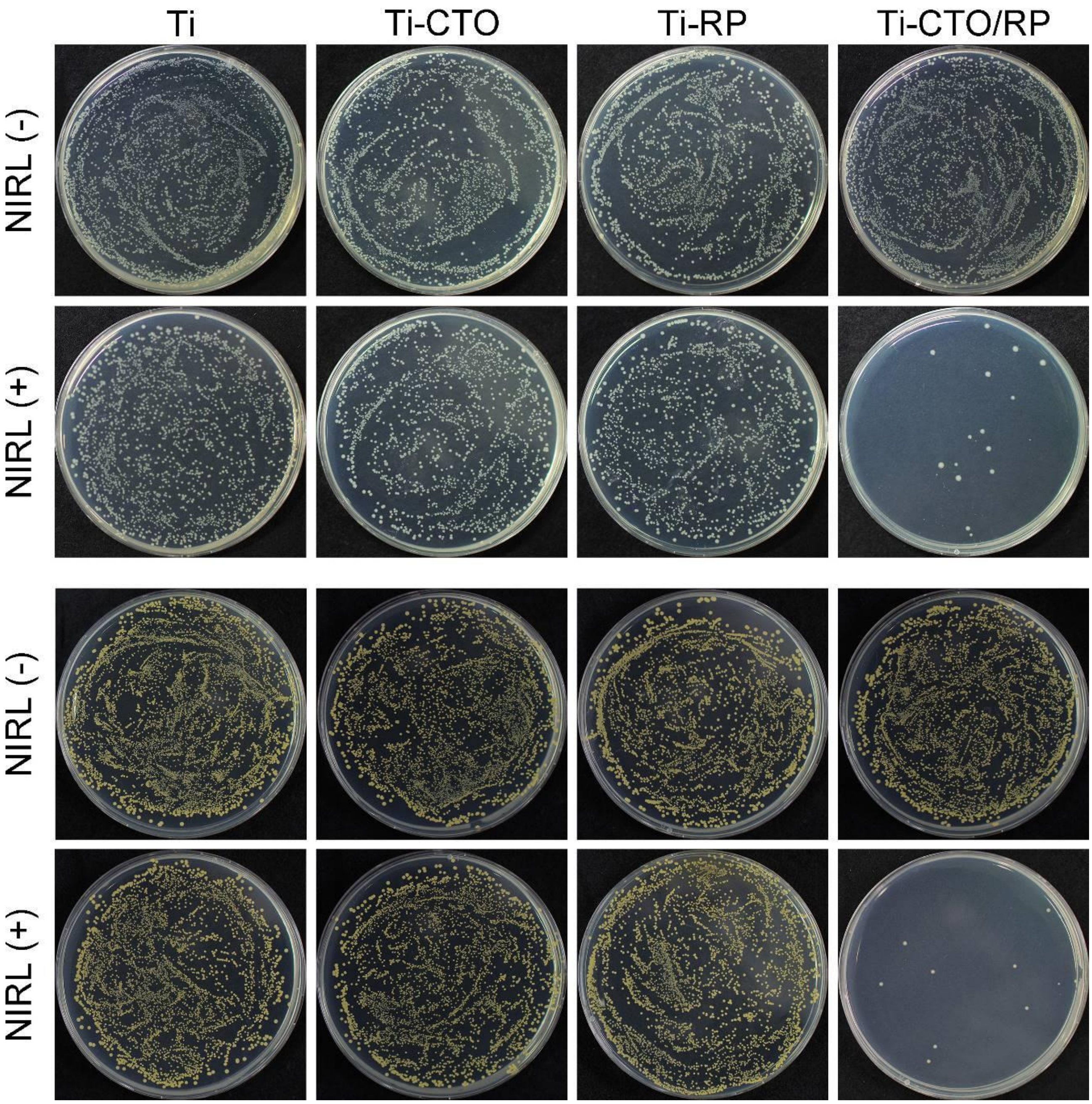

Figure S11. The visible colony units of *E. coli* (line 1 and 2) and MRSA (line 3 and 4) after treated with CTO/RP P-N heterojunction under the 808 nm NIRL irradiation or in the darkness compared with that of Ti, CTO and RP show the enhanced photocatalytic bacterial killing ability by CTO/RP heterojunction.

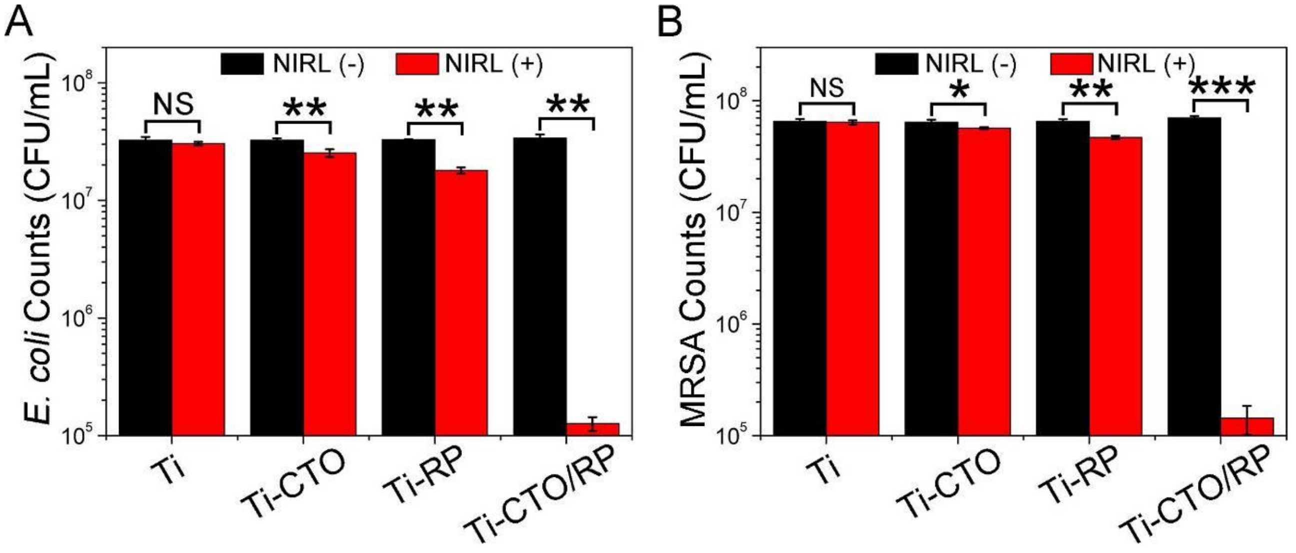

Figure S12. A, B) The CFU of A) *E. coli* and B) MRSA bacterial suspensions after treated with CTO/RP P-N heterojunction under the 808 nm NIRL irradiation or in the darkness compared with that of Ti, CTO and RP show the enhanced photocatalytic bacterial killing ability by CTO/RP heterojunction. Error bars indicate means ± standard deviations (n = 3): *$p < 0.05$, **$p < 0.01$, ***$p < 0.001$ (t-test). NS: not significant ($P > 0.05$).

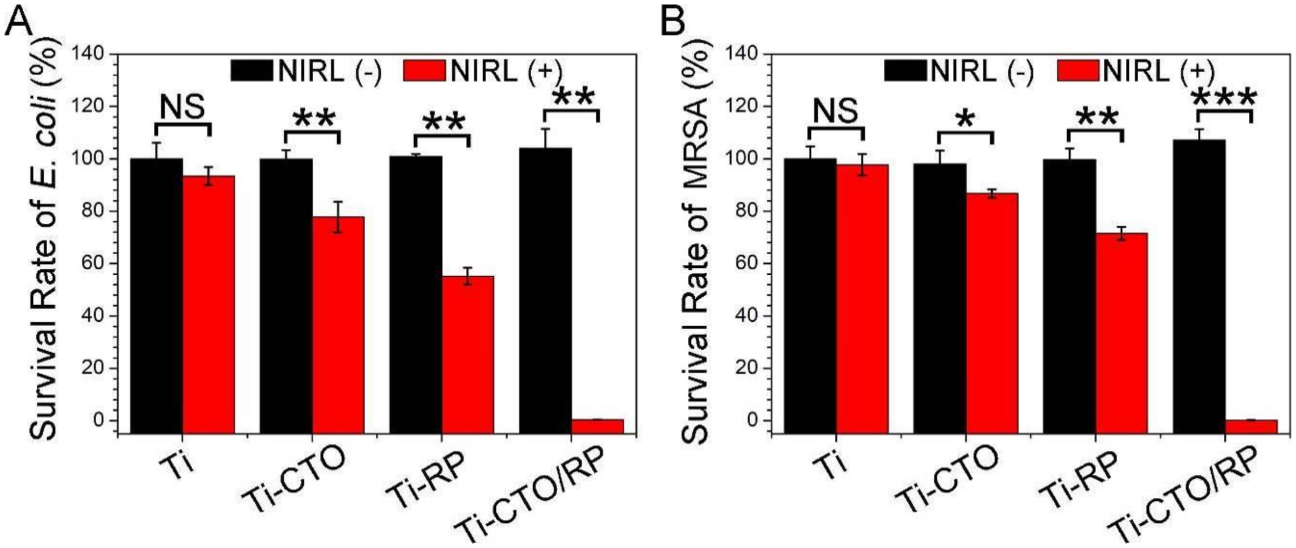

Figure S13. A, B) The survival ratios of A) *E. coli* and B) MRSA after treated with CTO/RP P-N heterojunction under the 808 nm NIRL irradiation or in the darkness compared with that of Ti, CTO and RP show the efficient bacterial killing ability by CTO/RP heterojunction after irradiation. Error bars indicate means ± standard deviations (n = 3): *$p < 0.05$, **$p < 0.01$, ***$p < 0.001$ (t-test). NS: not significant ($P > 0.05$).

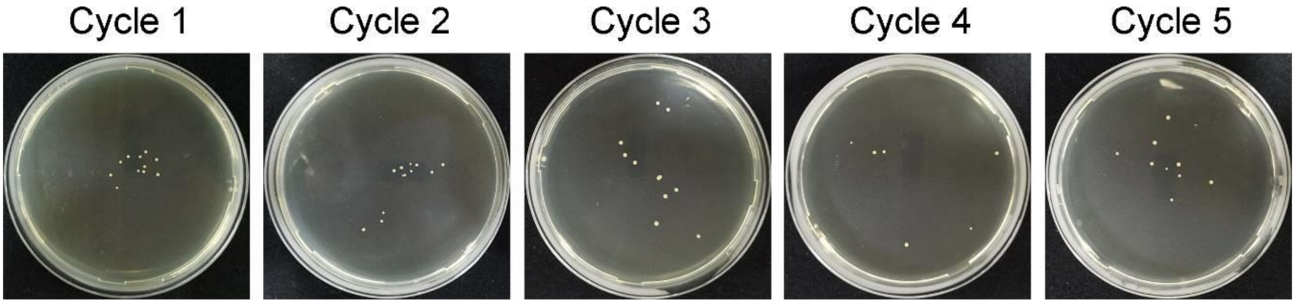

Figure S14. The visible colony units of MRSA after treated with the same CTO/RP sample under the 808 nm NIRL irradiation for 5 cycles.

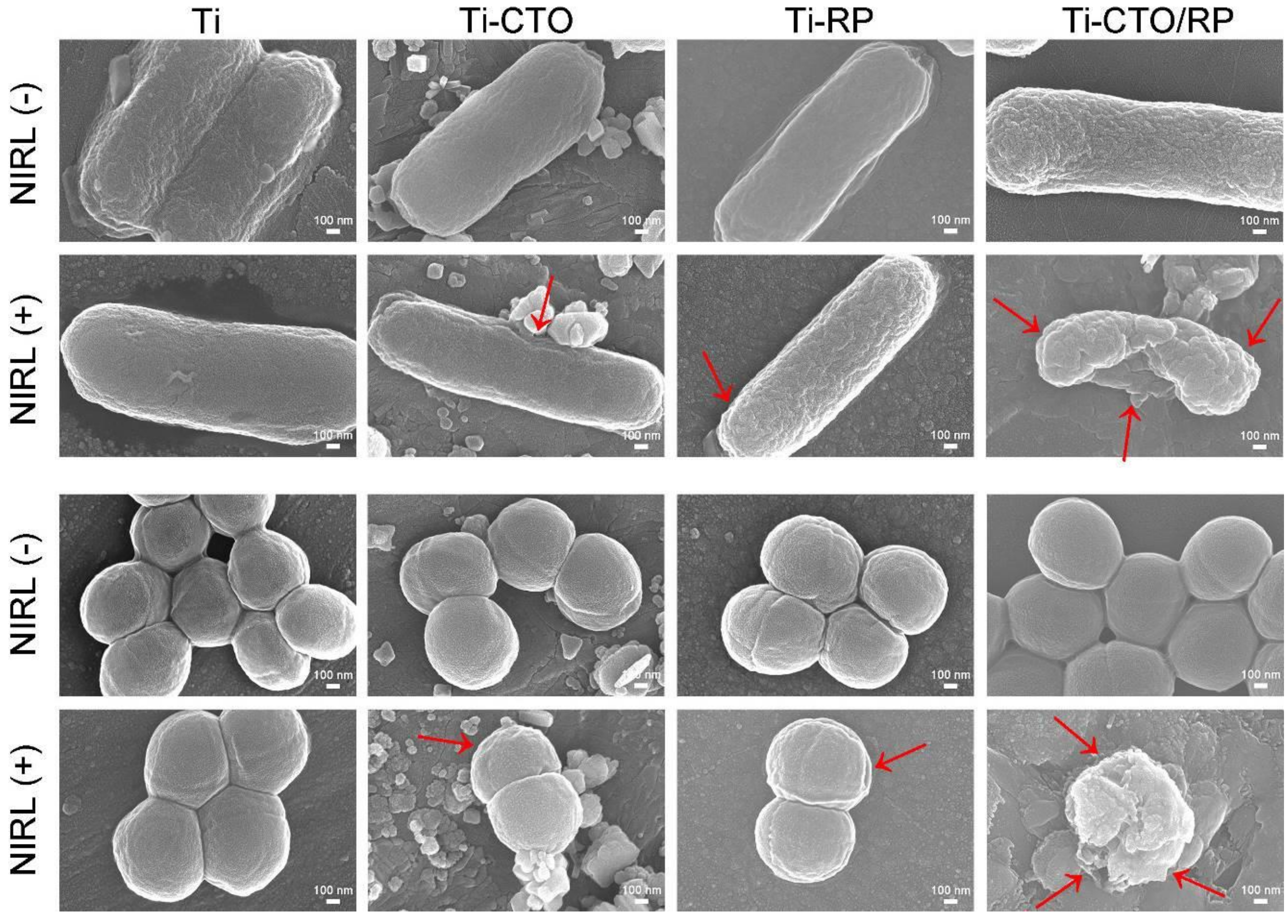

Figure S15. SEM morphologies of *E. coli* (line 1 and 2) and MRSA (line 3 and 4) after treated with CTO/RP P-N heterojunction under the 808 nm NIRL irradiation or in the darkness compared with that of Ti, CTO and RP show the serious damage to the bacteria by CTO/RP heterojunction after irradiation. Red arrows: damaged bacterial membranes. Scale bar: 100 nm.

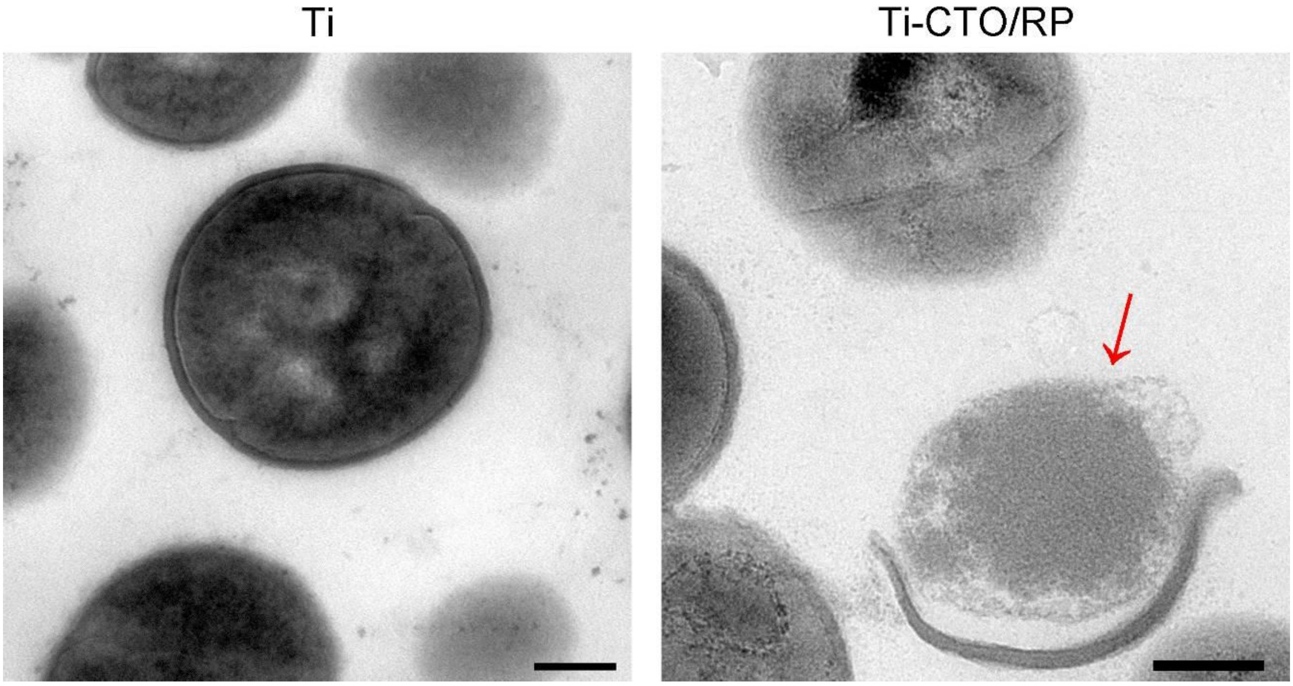

Figure S16. TEM morphologies of MRSA after treated with Ti and CTO/RP P-N heterojunction under the 808 nm NIRL irradiation show the serious damage to the MRSA by CTO/RP heterojunction after irradiation. Red arrows: damaged MRSA membranes. Scale bar: 200 nm.

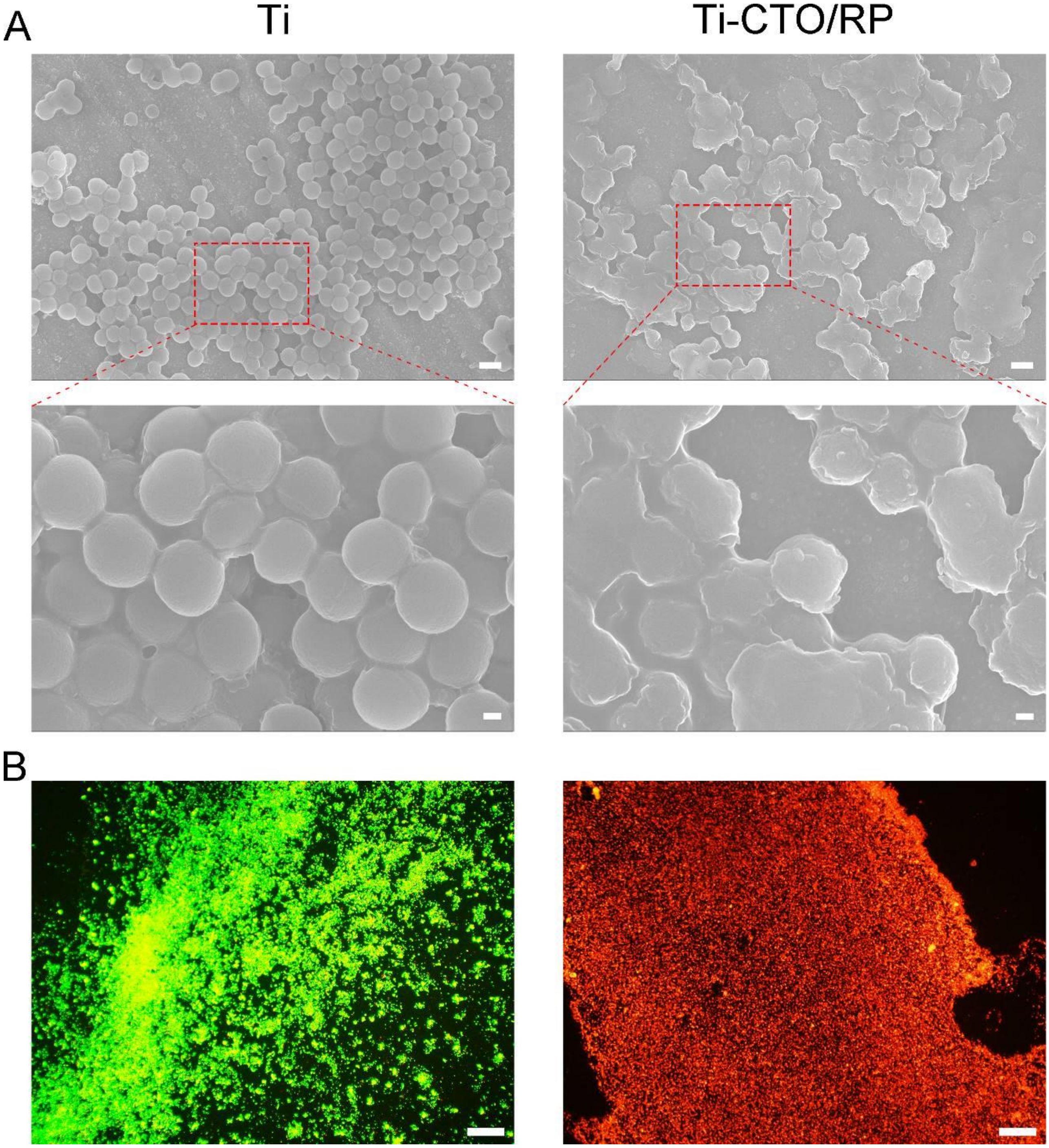

Figure S17. A) SEM morphologies of MRSA biofilm after treated with Ti and CTO/RP P-N heterojunction under the 808 nm NIRL irradiation show the serious damage to the MRSA biofilm by CTO/RP heterojunction after irradiation. Scale bar in low magnification images: 2 μm; Scale bar in high magnification images: 200 nm. B) Fluorescence images of MRSA biofilm, live MRSA was stained in green while dead MRSA was stained in red. Scale bar: 50 μm.

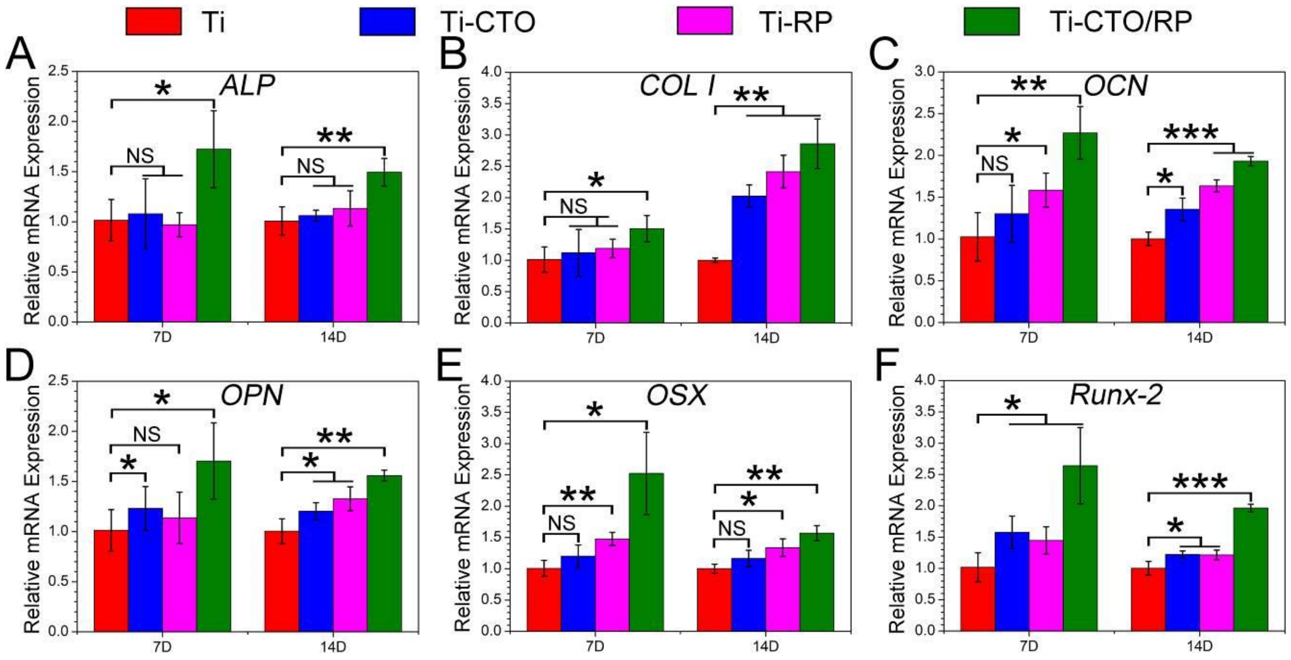

Figure S18. A-F) The quantification of A) *ALP*; B) *COL I*; C) *OCN*; D) *OPN*; E) *OSX*; and F) *Runx-2* in MSCs using GAPDH as a reference gene after cultured with Ti, CTO, RP and CTO/RP P-N heterojunction for 7 and 14 days. Error bars indicate means ± standard deviations (n = 3): *$p < 0.05$, **$p < 0.01$, ***$p < 0.001$ (t-test). NS: not significant ($P > 0.05$).

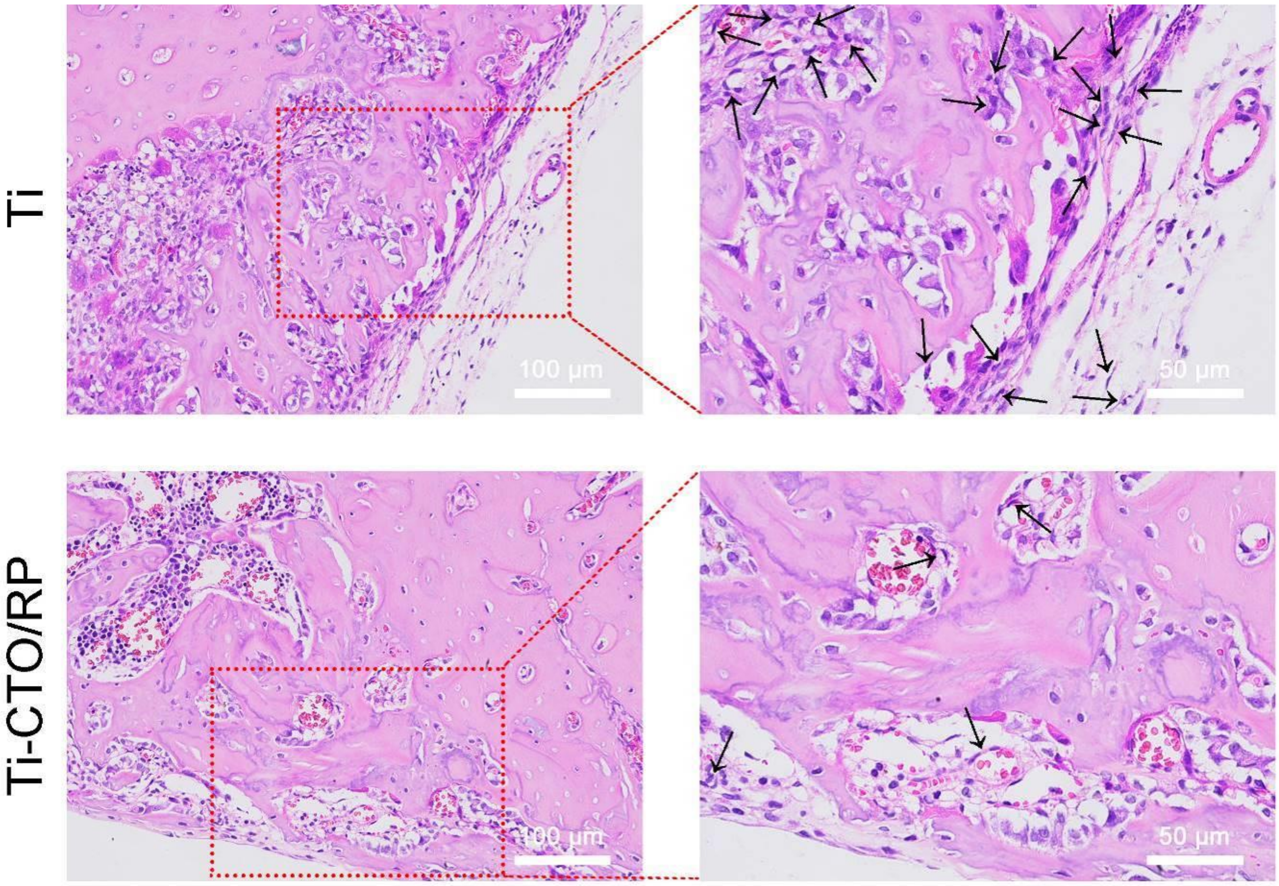

Figure S19. The histological images of the bone tissue samples taken from rats at the endpoint of 7 days and staining with hematoxylin and eosin (H&E). Scale bar in low magnification images (left): 100 μm; Scale bar in high magnification images (right): 50 μm. Black arrows: lobulated neutrophils.

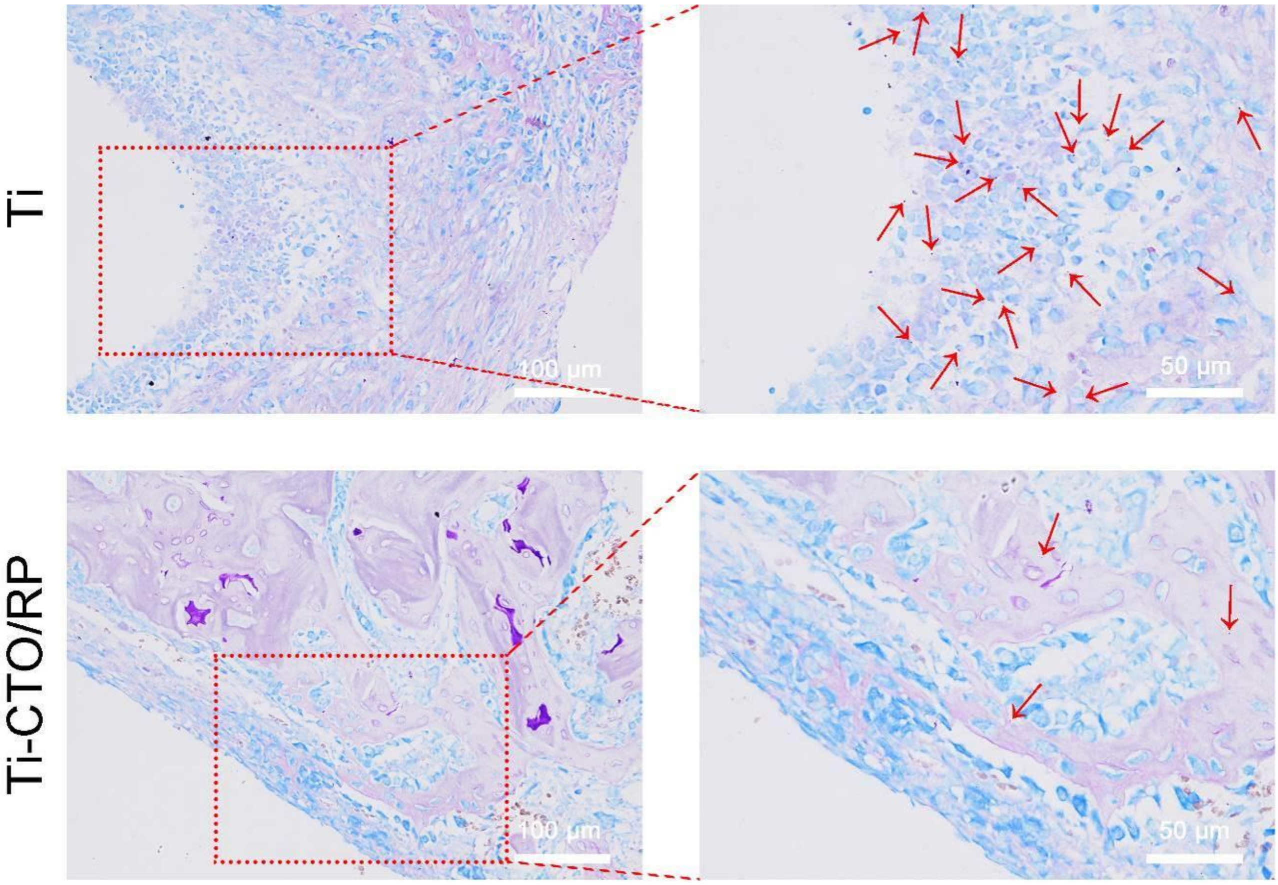

Figure S20. The histological images of the bone tissue samples taken from rats at the endpoint of 7 days and staining with Giemsa. Scale bar in low magnification images (left): 100 µm; Scale bar in high magnification images (right): 50 µm. Red arrows: bacteria.

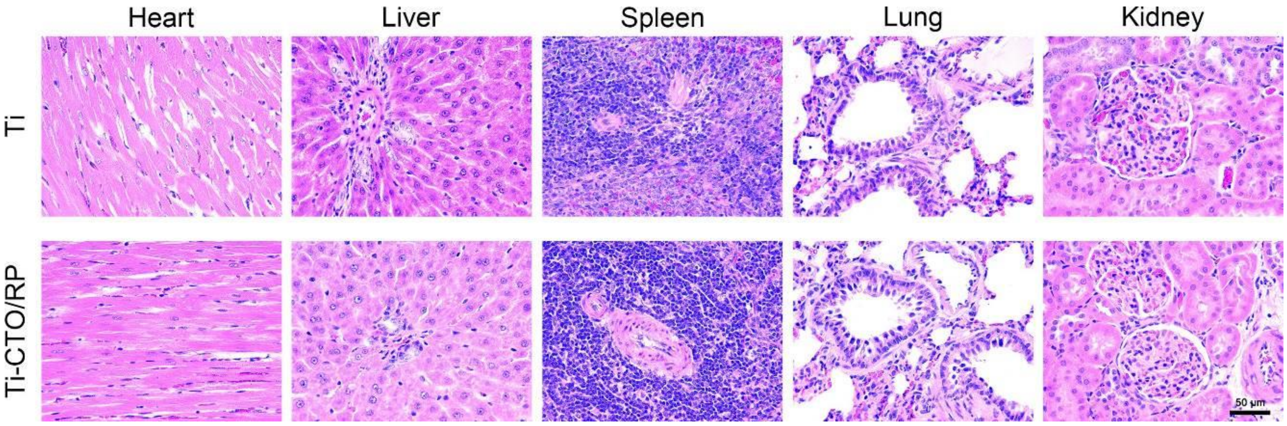

Figure S21. The histological images of the heart, liver, spleen, lung, and kidney tissue samples taken from rats at the endpoint of 4 weeks and staining with H&E. Scale bar: 50 μm.
